# Synoptic Overview of Exotic *Acacia*, *Senegalia* and *Vachellia* (Caesalpinioideae, Mimosoid Clade, Fabaceae) in Egypt

**DOI:** 10.3390/plants10071344

**Published:** 2021-07-01

**Authors:** Rania A. Hassan, Rim S. Hamdy

**Affiliations:** Botany and Microbiology Department, Faculty of Science, Cairo University, Giza 12613, Egypt; rhamdy@sci.cu.edu.eg

**Keywords:** *Acacia*, *Senegalia*, *Vachellia*, exotic species, taxonomy

## Abstract

For the first time, an updated checklist of *Acacia*, *Senegalia* and *Vachellia* species in Egypt is provided, focusing on the exotic species. Taking into consideration the retypification of genus *Acacia* ratified at the Melbourne International Botanical Congress (IBC, 2011), a process of reclassification has taken place worldwide in recent years. The review of *Acacia* and its segregates in Egypt became necessary in light of the available information cited in classical works during the last century. In Egypt, various taxa formerly placed in *Acacia s.l*., have been transferred to *Acacia* *s.s*., *Acaciella*, *Senegalia*, *Parasenegalia* and *Vachellia*. The present study is a contribution towards clarifying the nomenclatural status of all recorded species of *Acacia* and its segregate genera. This study recorded 144 taxa (125 species and 19 infraspecific taxa). Only 14 taxa (four species and 10 infraspecific taxa) are indigenous to Egypt (included now under *Senegalia* and *Vachellia*). The other 130 taxa had been introduced to Egypt during the last century. Out of the 130 taxa, 79 taxa have been recorded in literature. The focus of this study is the remaining 51 exotic taxa that have been traced as living species in Egyptian gardens or as herbarium specimens in Egyptian herbaria. The studied exotic taxa are accommodated under *Acacia s.s.* (24 taxa), *Senegalia* (14 taxa) and *Vachellia* (13 taxa). Identification keys for the studied genera, generic groups and species have been provided using different taxonomic criteria. For each taxon, the validated name with the first citation followed by relevant Egyptian citations, typification, synonyms, distinctive features, origin, ecology (when available), utilisation and selected specimens are provided. The study revealed the presence of 22 newly recorded taxa in Egypt. Additionally, a list of excluded, unvalidated and unresolved names is given.

## 1. Introduction

Fabaceae Lindl. or Leguminosae Juss. is the third largest Angiosperm family after Asteraceae Bercht. and J. Presl and Orchidaceae Juss., comprising approximately 770 genera and over 19,500 species [1,2,3]. Recent phylogenetic analysis [3] indicated that the classification of Fabaceae into three subfamilies (Caesalpinioideae DC., Mimosoideae DC., and Papilionoideae DC.) is obsolete, and recognized six subfamilies: Caesalpinioideae DC, Cercidoideae LPWG, Detarioideae Burmeist., Dialioideae LPWG, Duparquetioideae LPWG, and Papilionoideae DC. Currently, the subfamily Mimosoideae is recognized as a distinct clade, i.e., the mimosoid clade, nested within the subfamily Caesalpinioideae [3].

*Acacia* Mill. *s.l.* in its traditional circumscription is the second largest genus in Fabaceae (approximately 1503 species according to WorldWideWattle database [4]), widespread in tropical, subtropical and warm temperate areas of the world [1,5,6,7]. The majority of species are centered in Australia, many in America and Africa, and fewer in Asia [6,8,9].

This speciose genus has received considerable critical attention due to its biocultural, high species number, symbolic and economic significance in Africa, America and Australia [6]. Many of its species are dominant components of drier vegetation, such as the thorn scrubs of southern Africa and the mulga woodlands in Australia dominated by the mulga tree *Acacia aneura*.

The debate around the taxonomic status of the genus *Acacia* Mill. goes back a long way, with many taxonomists attempting to bring taxonomic order to this extremely diverse and species-rich group of mainly trees and shrubs [10]. Over the past two decades, there has been a dramatic increase in the number of *Acacia* species, and it became an urgent need to subdivide the genus into infrageneric groups [11,12,13,14].

In order to preserve the nomenclatural stability and conserve the genus name for the majority of *Acacia* species in Australia, [15,16] submitted a formal conservation proposal at the Vienna International Botanical Congress (2005) to re-typify the genus from its original African type species *A. scorpioides* (L.) W. Wight [now *A. nilotica* (L.) Willd. ex Delile] to the Australian type species *A. penninervis* Sieber ex DC. [synonymously *Racosperma penninerve* (DC.) Pedley] and was voted on. The acceptance of this retypification remained controversial [17,18,19,20,21,22,23]. Those opposing the retypification of *Acacia* argued that there were voting irregularities in Vienna leading up to Melbourne 2011.

At the XVIII meeting of International Botanical Code of nomenclature held in Melbourne (2011), *Acacia* was already in the draft Code as a conserved name, and the decision was to accept and ratify this Code [22,24,25]. In Appendix III (Nomina generica conservanda et rejicienda), *Acacia* is included with *A. penninervis* as a conserved type (Vienna Code, 2006: 286).

Molecular studies clearly indicated that *Acacia s.l.* is not monophyletic [7], and it should comprise at least seven genera [26], namely: *Acacia s.s.* (1082 species: 1077 in Australia,12 in Asia); *Acaciella* Britton and Rose (15 species in the Americas); *Mariosousa* Seigler and Ebinger (13 species in the Americas); *Parasenegalia* Seigler and Ebinger (11 species in the Americas); *Pseudosenegalia* Seigler and Ebinger (two species in the Americas); *Senegalia* Raf. (217 species: two species in Australia, 56 in Asia, 62 in Africa, 97 in the Americas); and *Vachellia* Wight and Arn. (164 species: nine species in Australia, 33 in Asia, 72 in Africa, 61 in the Americas), species numbers derived from the last update of WorldWideWattle website [4]. This new classification is based on results of many morphological, genetic and other studies during the last 50 years. As a result of the taxonomic fragmentation of *Acacia s.l.*, the names of many “*Acacia*” species worldwide have been changed in recent years. These names are accompanied by the names (now synonyms) that had been commonly used prior to fragmentation and retypification. 

Over the past decade, the definition of *Senegalia* has been refined. The small distinctive genus *Acaciella* that had been assigned to section or series Filicinae Benth. under both *Acacia* and *Senegalia* was resurrected by [27]. Three new genera were segregated from *Senegalia*, namely: *Mariosousa* [28], *Parasenegalia* and *Pseudosenegalia* [29].

Ref. [30] segregated the African species to either *Senegalia* or *Vachellia* using molecular data. These two genera are distinguished from each other by the presence of prickles and non-spiny stipules in *Senegalia*, as opposed to the absence of prickles and stipular spines found in *Vachellia*, among other characters [28]. New combinations were made in these genera with many authors [26,31,32,33].

In Egypt, the total native or indigenous species number is 1145 and 220 infraspecific taxa, and the family Leguminosae includes 228 species [34]. Various species formerly placed in *Acacia s.l*., have been transferred to *Acacia s.s.*, *Acaciella, Parasenegalia, Senegalia* and *Vachellia* (Appendix A and Appendix B). Collectively, these genera are called *Acacia s.l.* in the discussion below.

In Egyptian flora, *Acacia s.l.* appears to be the largest genus in the former subfamily Mimosoideae [34,35,36], represented by 14 indigenous taxa (included now under *Senegalia* and *Vachellia*). Many years ago, attempts were made to re-grow the native species in public and private Egyptian gardens, and in their natural areas as well, to preserve our genetic resources and restore the surrounding ecosystem, as many of these native species are facing threats of extinction in the wild due to water deficiency, global warming and habitat degradation.

On the other hand, the number of non-indigenous or exotic species which have been introduced to the country due to intentional or unintentional human involvement [37] is about 2000 species without succulents. The Leguminosae includes about 300 species [38,39,40].

By comparing the number of exotic or non indigenous species to native or indigenous species, which are almost similar. It is an equally important goal to carry out careful taxonomic revisions on the exotic species, updating their names and making an effort to define the unknown species, as many of them have been acclimatized and spread in the Egyptian gardens and streets, in addition to being used in many fields.

In earlier floristic studies, little attention was paid to exotic *Acacia s.l.* species in Egypt. During the reigns of Mohamed Ali, Ibrahim Pasha and Khedive Ismail (19th century), numerous *Acacia s.l.* species had been introduced and acclimatized over time in Egyptian gardens. More than 100 *Acacia* species were listed in a considerable amount of literature that dealt with the cultivated plants in Egypt (Appendix B). [38,41,42,43,44] are among the most important references. Despite this large number of recorded exotic *Acacia s.l.* species in Egypt, they lack satisfactory data concerning the precise description and real representation in the Egyptian herbaria.

The main goals of this study are to: provide a synopsis of the *Acacia, Senegalia and Vachellia* species that are introduced to the country; update the status of all recorded *Acacia* *s.l.* species in Egypt with the new generic classification and nomenclature; highlight the most important species that have been adapted to the Egyptian environment and are still growing; and provide full annotated appendices for the recorded indigenous and exotic *Acacia* *s.l.* species in Egypt (even species cited only in literature).

## 2. Results and Discussion

In this study, 144 *Acacia* taxa (125 species and 19 infraspecific taxa) have been recorded (Appendix A and Appendix B).

**Figure 1:**
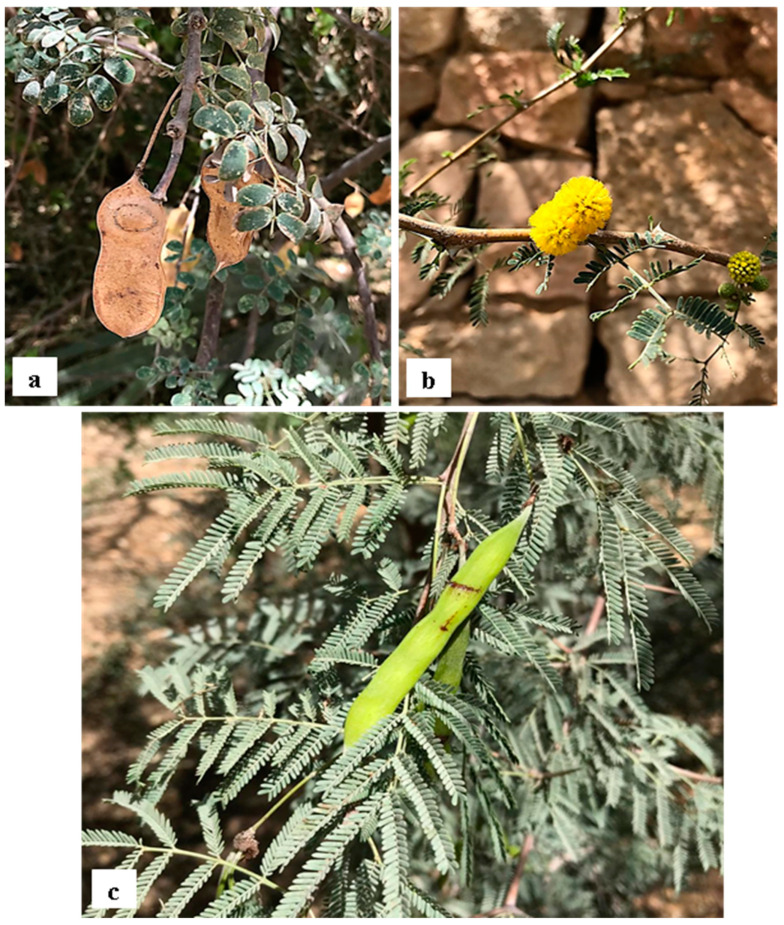
Examples of some indigenous species cultivated in a private botanical garden. (**a**) *Senegalia laeta*, bipinnate leaves and light brown pods, (**b**) *Vachellia* *seyal* var. *seyal*, bipinnate leaves with golden-yellow flowers in heads, (**c**) *Vachellia etbaica*, bipinnate leaves with immature pod.

**Figure 2:**
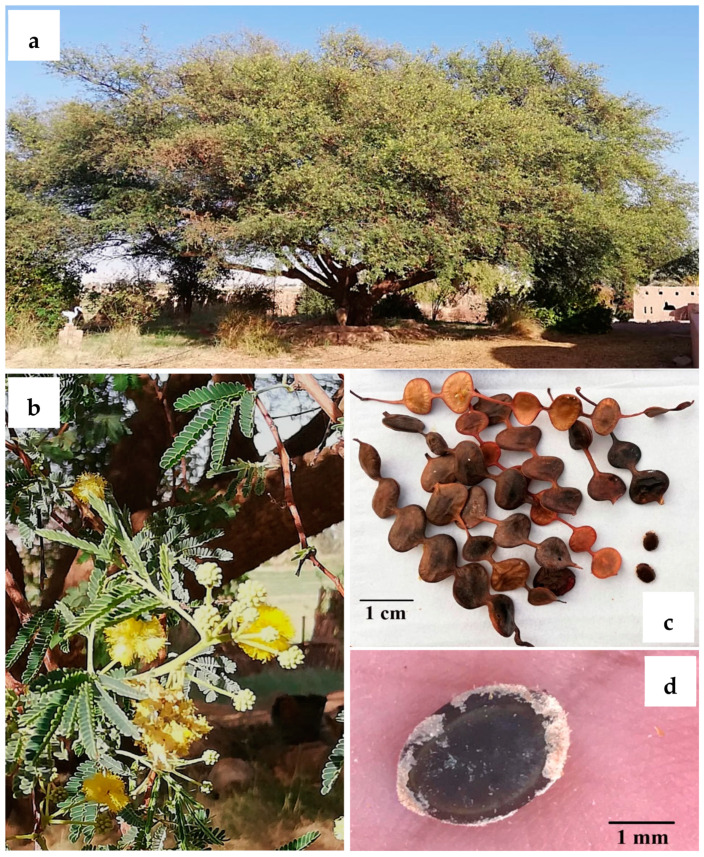
Example of an indigenous species growing in Dakhla Oasis. (**a**–**d**) *Vachellia nilotica* subsp. *nilotica,* (**a**) whole tree, (**b**) bipinnate leaves with golden yellow flowers in heads, (**c**) mature pods constricted between seeds, (**d**) dark brown seed.

Current evidence shows *Acacia s.l.* to be represented in Egypt by 14 indigenous taxa (four species, 10 infraspecific taxa; see Appendix A) placed in *Senegalia*, namely: *S. asak* (Forssk.) Kyal. and Boatwr.; *S. laeta* (R.Br. ex Benth.) Seigler and Ebinger [Figure 1a]; *S. mellifera* subsp. *mellifera* (Vahl) Seigler and Ebinger, and *Vachellia*, namely: *V. flava* (Forssk.) Kyal. and Boatwr.; *V. gerrardii* subsp. *negevensis* var. *najdensis* (Chaudhary) Ragup., Seigler, Ebinger and Maslin; *V. gerrardii* subsp. *negevensis* var. *negevensis* (Zohary) Ragup., Seigler, Ebinger and Maslin; *V. etbaica* (Schweinf.) Kyal. and Boatwr. [Figure 1c]; *V. nilotica* subsp. *adstringens* (Schumach. and Thonn.) Kyal. and Boatwr.; *V. nilotica* subsp. *nilotica* (L.) P.J.H. Hurter and Mabb. (Figure 2); *V. nilotica* subsp. *tomentosa* (Benth.) Kyal. and Boatwr.; *V. seyal* var. *seyal* (Delile) P.J.H.Hurter [Figure 1b]; *V. oerfota* var. *oerfota* (Forssk.) Kyal. and Boatwr.; *V. tortilis* subsp. *raddiana* (Savi) Kyal. and Boatwr.; *V. tortilis* subsp. *tortilis* (Forssk.) Galasso and Banfi.

*Acacia nilotica* (now *Vachellia nilotica*) has been known to the ancient Egyptians since pre-dynastic times, and the tree is well documented in inscriptions and wall paintings in tombs. Its wood was used for the manufacture of sarcophagi and agricultural tools, the pods were an essential source of tannins used for tanning and dyeing leather, and its thorns were used as needles.

On the other hand, 130 *Acacia s.l.* taxa were introduced to Egypt from different regions of the world during the last century to adorn our gardens and streets. The first effort was made in Egypt under the rule of Mohamed Ali Pasha, and it reached its zenith under the reign of Khedive Ismail. Some species have commenced to proliferate rapidly along North Sinai’s eastern and western coasts (e.g., *A. saligna*).

In terms of its economic significance in Egypt, the acacia tree was one of the most useful timber trees, with its sturdy, reddish-colored wood. It was used for making agricultural equipment, ships, furniture, sugar cane presses, ancient rice mills, car wheels as well as in buildings. It was also utilized as a source of fuel. The gum of *A. Senegal* exudate from the tree is used in medicine, while the flowers of *A. farnesina* were used for making perfumes and fragrant cosmetics. The higher nutritive value and production biomass of *A. saligna* make the genus an attractive animal fodder. *A. mangium*, considered to be an agroforestry tree, was introduced for its wood value and can provide some ecological services such as absorbing pollutants, sequestering carbon, providing shade, and creating oxygen.

All the exotic taxa recorded in Egypt are listed in Appendix B. Out of the 130 taxa, 79 taxa have been recorded in literature. The focus of this study is the remaining 51 exotic taxa (39 species, five subspecies, seven varieties).

The studied taxa are traced as living trees or shrubs growing in Egyptian gardens (36 taxa), preserved herbarium specimens (17 taxa) or newly recorded (23 taxa). The studied taxa are accommodated in the three new genera: *Acacia s.s*. (24 taxa), *Senegalia* (14 taxa), and *Vachellia* (13 taxa). Each of the studied genera is classified into a number of informal groups to facilitate the differentiation. An additional list of excluded, unvalidated and unresolved names is given. The identification keys of the studied genera and generic groups are based mainly on the leaf structure followed by the presence of spiny stipules or prickles and type of inflorescence.

Key to identify the studied genera
1A. Leaves reduced to phyllodes*Acacia s.s* (except sect. *Botrycephalae*)1B. Leaves bipinnate22A. Prickles present on branchlets and/or leaves; stipules not spinescent*Senegalia*2B. Prickles absent; stipules spinescent*Vachellia*

Taxonomic treatment

**A. *Acacia*** Mill., Gard. Dict. Abr., ed. 4, 1 (1754).

**Type species:***Acacia penninervis* Sieber ex DC., Prodr. 2: 452 (1825), type. cons.

Woody trees or shrubs, rarely lianas; branches rarely with prickles. Leaves bipinnate or modified to polymorphic phyllodes, rarely reduced to scales or absent; foliar glands normally present; stipules present, but commonly caducous and scarious, rarely spinose. Inflorescences simple, racemose or paniculate; flowers white to golden, rarely pink, 2 or more aggregated into globular heads or cylindrical spikes, (3–) 4 or 5 (–6)-merous; sepals free to united, rarely absent; stamens numerous, free, rarely united basally into a short tube; ovary sessile or rarely stipitate. Legumes dehiscent or rarely indehiscent.

According to WorldWide Wattle website [4], *Acacia s.s*. has 1080 species, mainly restricted to Australia (1075 in Australia,12 in Asia), making it the largest genus of vascular plants on that continent [9]. In Egypt, no native *Acacia s.s.* species are recorded. In the present study, 24 exotic taxa (23 species, one subspecies) are recognized. They are accommodated in six informal groups (A–F).

Key to identify the studied *Acacia* groups
1A. Leaves bipinnate, including adult stage**Group A**1B. Leaves reduced to phyllodes, bipinnate leaves found on seedlings, rarely persisting with phyllodes in adults22A. Flowers arranged in cylindrical spikes32B. Flowers arranged in globular heads43A. Phyllodes <10 mm wide, with obscured nerves**Group B**3B. Phyllodes >10 mm wide, with 3–4 (–5) conspicuous nerves**Group C**4A. Phyllodes with single central midrib54B. Phyllodes with (1) 2 or more longitudinal veins on at least one face**Group F**5A. Stipules spinescent**Group D**5B. Stipules not spinescent**Group E**

**Group A**: *Acacia* sect. *Botrycephalae* (Benth.) Taub. (1894).

**Type:** *Acacia botrycephala* (Vent.) Desf. (= *A. terminalis* (Salisb.) J.F. Macbr.)

Leaves bipinnate, including adult stage, stipules not spinescent, prickles absent.
1A. Interjugary glands absent21B. Interjugary glands present**3. *A. mearnsii***2A. Young foliage tips pale blue; leaflets (1–) 2–4 (–6) pairs per pinna**1. *A. baileyana***2B. Young foliage tips white, cream to golden; leaflets 6–30 pairs per pinna**2. *A. dealbata***2C. Young foliage tips silvery or yellowish-white; leaflets in 3–12 pairs per pinna**4. *A. pubescens***

**1. *Acacia baileyana*** F. Muell., Trans. Roy. Soc. Victoria 24 (2): 168 (1887-8). ≡ *Racosperma baileyanum* (F. Muell.) Pedley, Austrobaileya 2 (4): 345 (1987).

**Type:** Australia: Queensland, Brisbane, from a cultivated tree in Bowen’s Park, Aug.1876, flowering specimen given by *F. M. Bailey s.n.* (holotype: MEL-2078486! isotype: BRI-AQ0022725!). = *Acacia baileyana* var. *aurea* Pescott, Victorian Naturalist 34: 79 (1917).

**Distinctive features:** Shrub or tree up to 10 m high. Bark smooth, grey or brown. Young foliage-tips pale blue. Leaves subcoriaceous, blue-grey, glaucous; interjugary glands absent; pinnae (1–) 2–4 (–6) pairs. Inflorescences of axillary racemes 3–10 cm long, or terminal false-panicles. Heads globular, 11–25-flowered, golden. Pods 3–10 cm × 7.5–15 mm, coriaceous, bluish at first, later brown to black, glabrous.

Native to Australia (New South Wales). It was introduced to Egypt in the early 2000s [45].

**Ecology and utilisation:** It is regarded as a significant environmental weed in Victoria and other parts beyond its natural range, often escaping from gardens or landscape plantings into native ecosystems. It is also considered an invasive species in South Africa [46]. It is fast-growing, frost-resistant, and widely cultivated in parks, gardens and roadsides [47]. In Egypt, it is cultivated in some private gardens for its foliage and floral display.

**Selected specimens:** Giza: Maghraby garden, s.d., *T. Labib s.n.* (Orman Herbarium); Mazhar Botanic Garden, 15.01. 2019, *R. Hamdy 5011* (Cairo University, CAI).

**2. *Acacia dealbata*** Link, Enum. Hort. Berol. Alt. 2: 445 (1822); Delchev.Pl. Exo. Egypte: 3(1871); Delchev., Ap.Ge. végét. Exot. Égypte: 273 (1881); Delchev. Prom. et jard. Caire: 63 (1899); Asch. and Schweinf., Ill. Fl. Égypte, Mém. Inst. Égypt. 2: 72 (1887); Walsingham, Hort. Rev. 56:66 (1922). ≡ *Acacia decurrens* Willd. var. *dealbata* (Link) F. Muell. ex Maiden, Wattles and Wattle- barks, ed. 3: 39 (1906). *Racosperma dealbatum* (Link) Pedley, Austrobaileya 2: 358 (1987).

**Type:** British East Africa, 26 May-1 June 1909, *E.A.Mearns 249* (lectotype: BR-0000006251734!, designated by Brenan and Melville, 1960). = *Acacia decurrens* var. *mollis* Lindl., Bot. Reg. 5: t. 371 (1819). *Acacia puberula* Dehnh., Cat. Horti Camald., 2nd edn. 17 (1832).

**Distinctive features:** Bushy shrub or spreading tree up to 30 m high. Bark smooth, grey, brown or dark brown. Young foliage-tips white, cream-coloured to golden, velvety-tomentose. Leaves herbaceous, usually bluish grey or silvery and glaucous; interjugary glands absent; pinnae 6–30 pairs, 0.5–5.5 cm long. Inflorescences in axillary racemes, or mostly terminal or axillary false panicles. Heads globular, 13–42-flowered. Pods often slightly constricted between some or all seeds, 2–11.5 cm × 6–14 mm, subcoriaceous, blue or purplish, pruinose.

Native to Australia (Australian Capital Territory, New South Wales, Tasmania, Victoria). It was introduced to Egypt in the 19th Century [41,43].

**Ecology and utilisation:** It is considered a common environmental weed in South Australia [48]. It is widely cultivated and often an early coloniser after fire [49]. It is a fast-growing, nitrogen-fixing shrub or tree adapted to cool climates. It has an ornamental value and is used on farms for windbreaks and erosion control. The wood is excellent for pulping and is a satisfactory fuel [50]. According to [41,43,51], it is cultivated in many historical and public gardens in Cairo and Alexandria.

**Selected specimens:** Cairo: Manial Palace Garden, 4.03.1956; Maghraby Garden, 15.01. 2019, *R. Hamdy 5024* (CAI).

**3. *Acacia mearnsii*** De Wild., Pl. Bequaert. 3 (1): 61 (1925). ≡ *Racosperma mearnsii* (De Wild.) Pedley, Bot. J. Linn. Soc. 92 (3): 249 (1986).

**Type:** British East Africa: cultivated in Kenya, vicinity of Thika, 6–7 Sept. 1909, *E. A. Mearns 1092* (lectotype: BR-0000008909220!, designated by Brenan and Melville, 1960). = *Acacia decurrens* (J.C. Wendl.) Willd. var. *mollis* Lindl., Bot. Reg. 5: pl. 371 (1819).

**Distinctive features:** Spreading shrub or erect tree up to 10 (–16) m high. Bark smooth, corrugated at base when old, black or grey. Young foliage-tips yellow or greenish yellow, velvety-pubescent. Leaves bipinnate, subcoriaceous, dark green and glossy above; rachis 3–13.5 cm long, with 1 or 2 confluent interjugary glands between some or all pairs of pinnae; pinnae 7–31 pairs, 1.5–6 cm long. Inflorescences in axillary racemes, or terminal or axillary false panicles. Heads globular, 20–40-flowered, pale yellow or cream-coloured. Pods barely constricted between seeds, straight to curved, 3–15 cm × 4.5–8 mm, coriaceous, black, red-brown or dark brown, slightly scabrous.

Native to Australia (Australian Capital Territory, New South Wales, South Australia, Tasmania, Victoria). It was introduced to Egypt in the early 1950s [38].

**Ecology and utilisation:** It is widely cultivated in southern Australia and other parts of the world as an ornamental and forestry tree and readily escapes from cultivation. It is recorded as invasive according to the Global Invasive Species Database [52]. It is a serious invader in southern India and South Africa [53]. It is a multipurpose tree grown commercially for its wood and high tannin-yielding bark in Africa [54]. In Egypt, it is cultivated as an ornamental tree in some gardens.

**Selected specimens:** Giza: Mazhar Botanic Garden, 15.01.2019, *R. Hamdy 5041* (CAI).

**4. *Acacia pubescens*** (Vent.) R. Br. in W.T. Aiton, Hort. Kew., ed. 2, 5: 467 (1813); Delchev., Ap.Ge. végét. Exot. Égypte: 273 (1881). Basionym: *Mimosa pubescens* Vent., Jard. Malmaison 1: pl. 21 (1803); Delchev., Ap.Ge. Végét. Exot. Égypte: 272 (1881). ≡ *Racosperma pubescens* (Vent.) Pedley, Austrobaileya 6 (3): 482 (2003).

**Type:** Australia, Jardin Malmaison, Herbier *E.P. Ventenat s.n.* (holotype: G-00341443!). = *Acacia mollissima* Hort. Ex Willd., Enum. Pl. pt. 2: 1053 (1809).

**Distinctive features:** Weeping shrub 1–5 m high. Bark smooth, brownish grey. Young foliage-tips silvery or yellowish white, villous. Leaves herbaceous, bright green above, paler beneath, sometimes with a gland at basal pinnae; rachis 1.5–6.6 cm long, ridged, ±villous, mostly eglandular; pinnae 3–12 pairs, 0.5–2.5 cm long. Inflorescences in axillary racemes or terminal false panicles. Heads 8–16-flowered, golden. Pods 1.5–8 cm × 4–6.5 mm, subcoriaceous, bluish, bluish-brown or black, ± pruinose.

Endemic to Australia (New South Wales). It was introduced to Egypt in the 19th century [41].

**Ecology and utilisation:** It is a rare species, much of its original habitat in western Sydney has been destroyed for housing and other developments [55]. In Egypt, it is cultivated in historical gardens, producing flowers in clustered panicles with broadly linear hairless pods, and mentioned as *A. mollissima* by [41].

**Selected specimens:** Giza: Zoological Garden, 1.08.1968, *M. El Mahdi s.n.* (Orman Herbarium)-Ibid loc., 25.08.1968, *M. El Mahdi 5054* (CAI).

**Group B:** Leaves reduced to phyllodes, bipinnate leaves found on seedlings, rarely persist; flowers arranged in cylindrical spikes; phyllodes flat to terete, straight or curved to falcate, <10 mm wide, nerves obscure.

**5. *Acacia aneura*** F.Muell. ex Benth., Linnaea 26: 627 (1853); Walsingham, Hort. Rev. 56: 66 (1922). ≡ *Racosperma aneurum* (F. Muell. ex Benth.) Pedley, Austrobaileya 2: 344 (1987).

**Type:** Australia: South Australia, Prope Cudnaka, 1 Oct. 1851, *F. Muell. s.n.* (holotype: MEL- 724218! isotypes: PERTH-00600350!, PERTH-08247064!). = *Acacia aneura* var. *intermedia* Pedley, Fl. Australia 11B: 489 (2001).

**Distinctive features:** Shrub or tree 2.5–7 (−10 m) high. Phyllodes narrowly linear to linear-elliptic, shallowly incurved, flat, 4–12 (–18) cm × (1–) 1.5–3 (–4) mm, clustered in groups of 2 or 3 on juvenile plants, glabrous or obscurely appressed-hairy between veins, grey-green to subglaucous with a silvery sheen, longitudinal veins numerous and very fine. Inflorescence spicate; spikes (10–) 15–30 (–40) mm long. Pods straight-edged, flat, winged, chartaceous, brown to grey-brown, glabrous or sparsely appressed puberulous.

Notes: According to [56], species of the Mulga group (*A. aneura* and its close relatives) are enormously variable. Their identification in the field and from herbarium material is often challenging. Therefore, there is ambiguity concerning the application of the name *A. aneura* because of uncertainties regarding the taxon’s identity [57]. The taxonomic revision of Mulga group carried out by [56] in Western Australian defined 12 species based on morphological characters (branchlet resin; pod margins; phyllode shape, size, curvature; pod width).

Native to Australia (New South Wales, Northern Territory, Queensland, South Australia, Western Australia). It was introduced to Egypt in the early 1920s from Australia [44]. It was growing very well in the Egyptian environment and listed among the seeds available for exchange [58].

**Utilisation:** Seeds have been used as a food source by Aboriginal people. The wood has been used by indigenous people of the Pilbara for weapons, walking sticks and tapping sticks [59]. In Egypt, it is cultivated as an ornamental tree in gardens. It is considered among the best-known Australian acacias introduced to North Africa many years ago [60].

**Selected specimens:** Cairo, Zohriya Garden, 29.10.1929; *M. Hassib 5005* (CAI)-Giza: Cairo University Gardens, 29.10.1959, *V. Täckholm 5006* (CAI); Agricultural Museum, 20.03.1962, *M. Ezz Eldin s.n.* (Orman Herbarium)-Aswan: Aswan Botanic Island, 29.07.1971, *H. Kamel 5007* (CAI).

**Group C:** Leaves reduced to phyllodes, bipinnate leaves found on seedlings, rarely persist; flowers arranged in cylindrical spikes; phyllodes flat to terete, straight or curved to falcate, >10 mm wide, with 3–4 (5) conspicuous nerves.
1A. Flowers 4-merous**8. *A. longifolia* subsp. *sophorae***1B. Flowers 5-merous22A. Phyllodes prominently reticulate; minor nerves densely anastomosing and not parallel to major nerves**9. *A. mangium***2B. Phyllodes not prominently reticulate; minor nerves sparsely anastomosing and parallel to major nerves33A. Phyllodes with the major nerves neither running together nor confluent with one another at the base and not confluent with lower margin**6. *A. auriculiformis***3B. Phyllodes with some of or all the major nerves either running together or confluent with one another at the base and confluent with lower margin**7. *A. concurrens***

**Figure 3:**
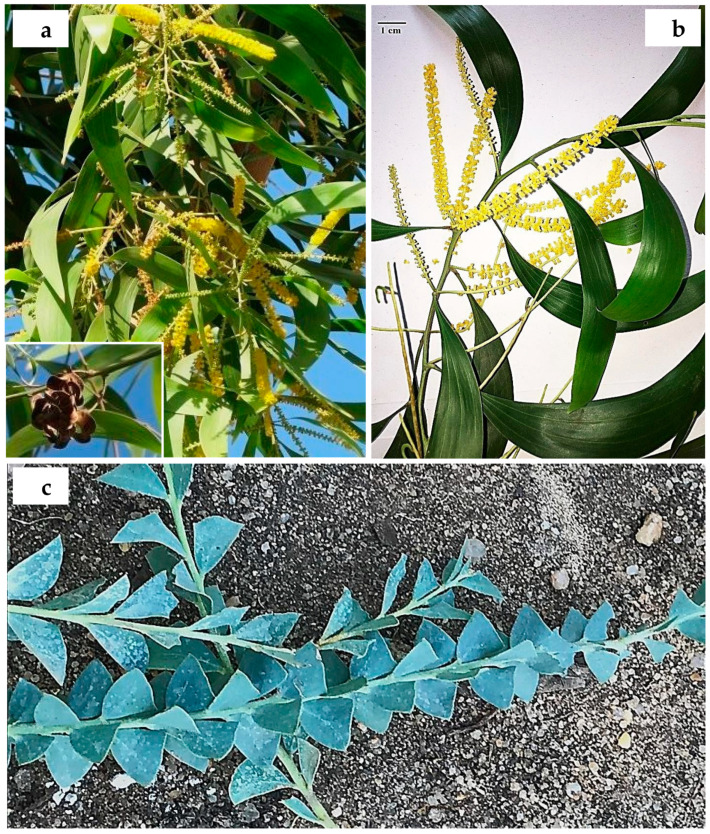
Examples of some studied *Acacia s.s.* species. (**a**,**b**) *Acacia auriculiformis,* leaves reduced to phyllodes with yellow flowers in spikes and brown coiled pods. (**c**) *Acacia cultriformis*, glaucous, inequilateral, triangular phyllodes.

**6. *Acacia auriculiformis*** A. Cunn. ex Benth., London J. Bot. 1: 377 (1842); Bircher, Gard. Hesperides: 337 (1960), [Figure 3a,b].

≡ *Racosperma auriculiforme* (A. Cunn. ex Benth.) Pedley, Bot. J. Linn. Soc. 92: 247 (1986).

**Type:** Australia: South Goulburn Island, Voyage of ‘Bathurst’, s.d., *A. Cunningham s.n.* (holotype: K-000793969!).

**Distinctive features:** Glabrous tree up to 35 m high. Bark fissured, grey to black. Phyllodes linear to very narrowly elliptic, falcate, (8–) 10–20 (–22) cm × (10–) 12–30 (–52) mm, with 3 subprominent longitudinal veins separate from one another until near base. Spikes 5–8.5 cm long, interrupted, yellow. Flowers 5-merous. Pods strongly curved to form an open coil, flat, outer margins undulate, 3.2–16 cm × 8–15 (–18) mm, coriaceous to ± woody, lightly pruinose, glabrous, transversely veined.

Native to Australia (Northern Territory, Queensland); Southeast Asia (Indonesia, West Papua, Moluccas, Papua New Guinea). It was introduced to Egypt in the early 1950s [38].

**Ecology and utilisation:** Its rapid growth rate, ability to fix nitrogen, tolerance to variable soil types make it a beneficial species for rehabilitation of degraded lands. It has been widely planted for fuelwood production, erosion control, ornament or shade mainly in Asia, Africa and South America. The wood is suitable for construction works (e.g., framing, flooring), woodturning and carving. The bark tannins are used for commercial exploitation [50]. In Egypt, it is cultivated as an ornamental tree with its showy flowers in some gardens.

**Selected specimens:** Giza, Mazhar Botanic Garden, 15.01.2019, *R. Hamdy 5010* (CAI).

**7. *Acacia concurrens*** Pedley, Contr. Queensland Herb. 15: 9 (1974). ≡ *Racosperma concurrens* (Pedley) Pedley, Bot. J. Linn. Soc. 92: 248 (1986).

**Type:** Australia: near Brisbane River, in forest ground, Queensland, Herb. Hookerianum 1867, s.d., *A. Cunningham s.n.* (holotype: K-000821200!).

**Distinctive features:** Shrub or tree up to 10 m high. Bark longitudinally fissured, fibrous, grey-black. Phyllodes oblique, narrowly elliptic, flat, with upper margin curved and lower margin straight, 8–16 (–18) cm × (9–) 12–35 (–60) mm, with (2–) 3–4 (–5) longitudinal veins (lower 2 confluent with each other near base). Spikes 3.5–11 cm long, pale yellow. Flowers 5-merous. Pods linear, slightly moniliform, semicircular, 5–10 cm long.

Native to Australia (New South Wales, Queensland). It was introduced to Egypt in the 2000s [45].

**Utilisation:** It has potential for use as a stock food during drought, and is useful for site rehabilitation after sand mining [50]. In Egypt, it is cultivated as an ornamental tree in some gardens.

**Selected specimens:** Giza, Mazhar Botanic Garden, 15.01.2019, *R. Hamdy 5020* (CAI).

**8. *Acacia longifolia*** subsp. *sophorae* (Labill.) Court, Fl. Australia 11B: 491 (2001).

Basionym: *Mimosa sophorae* Labill., Nov. Holl. Pl. 2: 87, pl. 237 (1807). ≡ *Acacia sophorae* (Labill.) R.Br., Hortus Kew. ed. 2, 5: 462 (1813); Delchev. Pl. Exo. Egypte: 4 (1871); Delchev., Ap.Ge. végét. Exot. Égypte: 273 (1881). *Phyllodoce sophora* (Labill.) Link, Handbuch 2: 133 (1831). *Cuparilla sophorae* (Labill.) Raf., Sylva Tellur. 120 (1838). *Acacia longifolia* var. *sophorae* (Labill.) Benth., Fl. Australia 2: 398 (1864).

**Type:** Australia: coll. unknown “protologue locality Capite Van-Diemen” [= Tasmania], [?J. J. H. de Labillardière], Herb. Ventenat (lectotype, BM-000833258! designated by Pedley, 1978).

**Distinctive features:** Shrub up to 5 m high, to 15 m or more wide, spreading, tangled, rarely erect. Phyllodes elliptic to narrowly elliptical (sometimes obovate to oblanceolate), 5–12 cm × 10–30 mm, obtuse or sometimes with a small mucronate point, subcoriaceous, sometimes fleshy, yellowish green. Inflorescence spicate. Flowers 4-merous. Pods coiled or contorted, firmly coriaceous.

Native to Australia (Queensland, South Australia, Tasmania, Victoria). It was introduced to Egypt in the 19th century, under the name *A. sophorae* [41].

**Ecology and utilisation:** It is known for its nitrogen-fixing ability, so used in coastal dune restoration and rehabilitation. It is recorded as invasive according to the Global Invasive Species Database [52]. In Victoria and South Australia, it has become a serious environmental weed of near-coastal heaths and woodlands. It has also become a very serious problem within its geographic native range in the last two decades [61,62]. In Egypt, it is cultivated as an ornamental tree in historical gardens.

**Selected specimens:** Giza, Mazhar Botanic Garden, 15.01.2019, *R. Hamdy 5038* (CAI).

**9. *Acacia mangium*** Willd., Sp. Pl. ed. 4, 4(2): 1053 (1806). ≡ *Racosperma mangium* (Willd.) Pedley, Austrobaileya 2: 352 (1987).

**Type:** Eastern Indonesia: Moluccas, based on description and figure in Rumphius, Herb. Amboin. 3: 123, pl. 81 (1750). = *Mangium montanum* Rumph., Herb. Amboin. 3: 123, t. 81 (1743).

**Distinctive features:** Tree 7–30 m high. Bark corrugated or coarsely cracked, grey to dark brown. Phyllodes narrowly elliptic to elliptic, 11–27 cm × 23–95 mm, coriaceous or papery, glabrous, with 3 or 4 (–5) main prominent nerves (confluent at base of phyllodes near lower margin). Spikes 5–12 cm long, loosely arranged, white to cream-coloured. Flowers 5-merous. Pods linear, openly coiled and twisted or tightly spirally coiled, 3–5.5 mm wide, coriaceous to subwoody, glabrous.

Native to Australia (Queensland); Southeast Asia (Indonesia, Papua New Guinea). It was introduced to Egypt in the 2000s [63].

**Ecology and utilisation:** It is recorded as invasive according to the Global Invasive Species Database [52]. It is a significant reforestation species in tropical Asia. The leaves can serve as forage for livestock. Heartwood is suitable for furniture, cabinet making, agricultural tools, boxes and crates, while mature wood is used as a good fuel [50]. In Egypt, the tree has an excellent potential to restore soil fertility, so it is currently cultivated in agroforestry systems in Serapium forest (Ismailia) for its wood and in some gardens as an ornamental tree.

**Selected specimens:** Giza: Mazhar Botanic Garden, 15.01.2019, *R. Hamdy 5040* (CAI).

**Group D:** Leaves reduced to phyllodes, bipinnate leaves found on seedlings, rarely persist; flowers arranged in globular heads; phyllodes with central midrib; stipules spinescent.
-Inflorescence simple, usually one per axil; peduncles not subtended by a secondary phyllode**10. *A. paradoxa***-Inflorescence racemose; peduncles sometimes subtended by a secondary phyllode**11. *A. victoriae***

**10. *Acacia paradoxa*** DC., Cat. Pl. Horti Monsp.: 74 (February–March 1813). ≡ *Racosperma paradoxum* (DC.) Pedley, Austrobaileya 6 (3): 479 (2003).

**Type:** France: cultivated in Montpellier botanic garden, 1812 (holotype: G-DC). *= Acacia armata* R.Br., in W.T. Aiton, Hort. Kew., ed. 2, 5: 463 (1813); Delchev.Pl. Exo. Egypte: 3(1871); Delchev., Ap.Ge. végét. Exot. Égypte: 273 (1881); Delchev. Prom. et jard. Caire: 63 (1899); Walsingham, Hort. Rev. 56: 106 (1922).

**Distinctive features:** Shrub or tree 2–5 (–8) m high. Stipules spinose, spreading, 4–15 mm long. Phyllodes erect, oblique or dimidiate, lanceolate, sometimes narrowly oblong-elliptic, 8–20 × 2–7 (–11) mm, acute or obtuse, glabrous to sparsely hairy; midrib normally excentric; abaxial margin normally undulate. Inflorescences simple, 1 per axil; heads globular, (20–) 30–50-flowered, golden. Pods linear to narrowly oblong, up to 6 cm long, 3–5 mm wide, thinly coriaceous, densely hairy.

Native to Australia (Australian Capital Territory, New South Wales, Queensland, South Australia). It was introduced to Egypt in the 19th century, under the name *A. armata* [41,43].

**Ecology and utilisation:** It is regarded as an environmental weed in Tasmania and Western Australia. It has become naturalised outside its native range in California, and is classified as a noxious weed in this state. It has been planted as a hedge [64,65]. In Egypt, it is cultivated as an ornamental tree in historical gardens.

**Selected specimens:** Giza, Maghraby Garden, 1.04.2009; *T. Labib s.n.* (Orman Herbarium).

**11. *Acacia victoriae*** Benth., in T.L. Mitchel, J. Exped. Trop. Australia 333 (1848). ≡ *Acacia sentis* var. *victoriae* (Benth.) Domin, Biblioth. Bot. 89: 254 (1926). *Racosperma victoriae* (Benth.) Pedley, Bot. J. Linn. Soc. 92: 249 (1986).

**Type:** Australia: Victoria River, New Holland, Queensland, 1 Oct. 1846, *T.L. Mitchell 620* (holotype: K-000791809!). = *Acacia hanniana* Domin, Biblioth. Bot. 89: 253 (1926). *Acacia coronalis* J.M. Black, Trans. Roy. Soc. S. Australia 71: 20 (1947).

**Distinctive features:** Shrub or tree 2–5 (–9) m high. Stipules spinose, 2–12 mm long. Phyllodes variable, linear to narrowly oblong, lanceolate or narrowly elliptic, straight or incurved, (1.4−) 2–5 (–10) cm × 2–8 mm, green to grey-green or glaucous, usually glabrous; midrib prominent; lateral veins usually obscure. Inflorescences racemose; peduncles sometimes subtended by a secondary phyllode; heads prolific, globular, 15–30-flowered, creamy white to pale lemon-yellow. Pods narrowly oblong, up to 8 cm long, 9–16 mm wide, chartaceous, glabrous.

Native to Australia (New South Wales, Northern Territory, Queensland, South Australia, Victoria, Western Australia). It was introduced into Egypt in the 2000s [45].

**Utilisation:** It is a useful stock food supplement during droughts and for soil stabilisation in dry countries. It also contains compounds called avicins, which may have medicinal uses [66]. It has been successfully used in Israel and Libya under 150–200 mm of rainfall. It also has a feed value comparable to *A. saligna* [60]. In Egypt, it is cultivated as an ornamental tree in some private gardens.

**Selected specimens:** Giza, Mazhar Botanic Garden, 15.01.2019, *R. Hamdy 5063* (CAI).

**Group E:** Leaves reduced to phyllodes, bipinnate leaves found on seedlings, rarely persist; flowers arranged in globular heads; phyllodes with central midrib; stipules not spinescent.
1A. Phyllodes ≤3.5 cm long21B. Phyllodes >3.5 cm long52A. Branchlets hairy to some degree**20. *A. vestita***2B. Branchlets completely glabrous33A. Heads with 3–12 flowers**13. *A. fimbriata***3B. Heads with more than 12 flowers44A. Phyllodes symmetrical with both lower and upper margins curved**15. *A. podalyriifolia***4B. Phyllodes asymmetric with lower margin straight and upper margin angled**12. *A. cultriformis***5A. Racemes enclosed (when young) by a series of small or large, caducous, imbricate bracts 65B. Racemes ebracteate at the base, or with a single small bract76A. Heads with 15–25 cream to pale yellow flowers**18. *A. salicina***6B. Heads with 25–78 golden flowers**19. *A. saligna***7A. Phyllodes 1–5 mm wide**16. *A. pycnantha***7B. Phyllodes >5 mm wide88A. Mature phyllodes appressed hairy**14. *A. neriifolia***8B. Mature phyllodes glabrous**17. *A. retinodes***

**12. *Acacia cultriformis*** A. Cunn. ex G. Don in Gen. Hist. 2: 406 (1832); Delchev. Pl. Exo. Egypte: 3(1871); Delchev., Ap.Ge. végét. Exot. Égypte: 273 (1881); Delchev. Prom. et jard. Caire: 63 (1899), [Figure 3c]. ≡ *Racosperma cultriforme* (A. Cunn. ex G. Don) Pedley, Austrobaileya 2: 347 (1987).

**Type:** Australia, Hunter River, New South Wales, Aug. 1827, *A. Cunningham 73* (holotype: K; isotype: BM-000796757!). *= Acacia scapuliformis* A. Cunn. ex G. Don, Gen. Hist. 2: 405 (1832). *Acacia cultrata* Paxton, Paxton’s Mag. Bot. 6: 259 (1839).

**Distinctive features:** Shrub up to 4 m high. Branchlets completely glabrous. Phyllodes crowded, inequilateral, often ±triangular, with abaxial margin ±straight and adaxial margin markedly rounded or angled, 10–30 × 5–15 mm, coriaceous, grey-green to glaucous, glabrous; midrib central or excentric. Racemes prolific in upper axils; axes 1–5 cm long, glabrous; heads globular to shortly cylindrical, 13–40-flowered, bright golden. Pods narrowly oblong, up to 9 cm long, (4–) 5–8 mm wide, chartaceous, often pruinose, glabrous.

Native to Australia (New South Wales, Queensland). It was introduced to Egypt in the 19th century, under the name *A. armata* [41,43] and listed among the seeds available for exchange [58].

**Ecology and utilisation:** It is regarded as an environmental weed in New South Wales outside its native range [67]. In Egypt, it is cultivated as an ornamental tree in historical and some private gardens and listed among the seeds available for exchange (Anonymous, 1922).

**Selected specimens:** Giza, Maghraby Garden, 05.2005; *T. Labib s.n.* (Orman Herbarium).

**13. *Acacia fimbriata*** A. Cunn. ex G. Don, Gen. Hist. 2: 406 (1832). ≡ *Acacia prominens* var. *fimbriata* (A. Cunn. ex G. Don) Domin, Biblioth. Bot. 89: 256 (1926).

*Racosperma fimbriatum* (A. Cunn. ex G. Don) Pedley, Austrobaileya 2(4): 348 (1987).

**Type:** Australia: Queensland, Brisbane River, September 1828, *A. Cunningham 158* (holotype: BM000796669!). *= Acacia prominens* var. *whiteana* Domin, Biblioth. Bot. 89: 256 (1926).

**Distinctive features:** Shrub or tree up to 6 m high. Branchlets completely glabrous. Phyllodes linear to narrowly oblong-elliptic or narrowly lanceolate, 2–5 cm × 2–5 mm, acute to obtuse-mucronulate, sparsely to densely fimbriolate with appressed hairs, sometimes glabrous; midrib fine. Inflorescences racemose; raceme axes 1.5–7.5 cm long, slender, glabrous or hairy; heads showy, globular, 3–12-flowered, bright golden. Pods up to 8 cm long, 5–9 mm wide, chartaceous, commonly pruinose, glabrous.

Native to Australia (New South Wales, Queensland). It was introduced to Egypt in the 20th century

**Utilisation:** In Egypt, it had been cultivated in botanical gardens as an ornamental tree in the past.

**Selected specimens:** Giza, Faculty of Agriculture, 20.08.1968, *M. Ezz El Din* *and B. Diwan* (Orman Herbarium)- ibid. Loc., 20.08.1974, *B. Diwan s.n.* (Orman Herbarium).

**14. *Acacia neriifolia*** A. Cunn. ex Benth., London J. Bot. 1: 357 (1842); Walsingham, Hort. Rev. 56:106 (1922); Bircher, Gard. Hesperides: 339 (1960). ≡ *Racosperma neriifolium* (A. Cunn. ex Benth.) Pedley, Austrobaileya 2: 353 (1987).

**Type:** Australia: New South Wales, summit of Mountain Hoddle, Liverpool Plains, May 1825, *A. Cunningham 101* (isosyntype: GH-00058353!), Austrobaileya 1: 289 (1980)- Ibid Loc., 1862, *C. Fraser s.n.* (isolectoype: BM-000810727!); *F. Bauer s.n.* (paralectotype: K-000791847!). *= Acacia penninervis* var. *angustata* F.M. Bailey, Syn. Queensl. Fl. 136 (1883).

**Distinctive features:** Shrub or tree up to 9 m high. Branchlets completely glabrous. Phyllodes narrowly elliptic to linear, straight or recurved, 6–15 cm × 4–9 mm. Inflorescences racemose; raceme axes 3–6 cm long, slender, appressed-puberulous with white or pale yellow hairs; heads showy, globular, 20–40-flowered, vivid golden. Pods up to 15 cm long, 5–10 mm wide, coriaceous, glabrous.

Native to Australia (New South Wales, Queensland). It was introduced to Egypt in the 20th century, under the name Bald Acacia, from Australia [38].

**Utilisation:** It is cultivated in eastern Australia as an attractive ornamental tree, sometimes grown as windbreaks. It has some value as forage for animals during times of drought. The bark has a tannin content and was used locally for tanning in southern Queensland [50]. In Egypt, it is cultivated as an ornamental tree in some botanical gardens.

**Selected specimens:** Cairo, Zohriya Garden, 1.05.1923, *J. Shabetai D2231* (Agricultural Research Center, CAIM); Manial Garden, Rhoda, 11.02.1968, *M. El Mahdi 5047* (CAI).

**15. *Acacia podalyriifolia*** A. Cunn. ex G. Don, Gen. Hist. 2: 405 (1832) as *podalyriaefolia*; Walsingham, Hort. Rev. 56: 106 (1922); Bircher, Gard. Hesperides: 340 (1960). ≡ *Racosperma podalyriifolium* (A. Cunn. ex G. Don) Pedley, Austrobaileya 2 (4): 354 (1987).

**Type:** Australia: Queensland, Birnam Range, Brisbane River, July 1828, *A*. *Cunningham 157* (holotype: K-00080603; isotype: BM-000796769!). *= Acacia fraseri* Hook., Icon. Pl. 2: pl. 171 (1837). *Acacia deneufvillei* L.Winter ex A. Berger, Gartenwelt 14: 112 (1910). *Acacia hanburyana* L. Winter ex A. Berger, Gartenwelt 14: 111 (1910).

**Distinctive features:** Spreading tree 3–7 m high. Branchlets completely glabrous. Phyllodes elliptic, oblong-elliptic, ovate or sometimes obovate, 2–4 (–6) × 1–2.5 (–3) cm, mucronate, silvery grey to glaucous, with slightly excentric midrib, finely penninerved. Inflorescence racemose; raceme axes 2–11 cm long; heads showy, fragrant, globular, 15–30-flowered, bright light golden. Pods up to 12 cm long, 1.5–2 cm wide, coriaceous, velvety and pruinose when young, glabrous with age, dehiscing unilaterally; margins undulate.

Native to Australia (New South Wales, Queensland). It was introduced to Egypt in the 20th century, under the name Pearl Acacia, from Australia [38].

**Ecology and utilisation:** It is regarded as an environmental weed in New South Wales, Victoria, South and Western Australia. It is currently widespread and has become naturalised beyond its native range. It is regarded as a ‘potential transformer’ in South Africa where it can replace indigenous species of natural vegetation. It is widely cultivated for decorative purposes [68,69]. In Egypt, it is cultivated as an ornamental tree in some gardens.

**Selected specimens:** Giza, Maghraby Garden, 09.2010, *T. Labib s.n.* (MAZHAR).

**16. *Acacia pycnantha*** Benth., London J. Bot. 1: 351 (1842); Walsingham, Hort. Rev. 56: 106 (1922). ≡ *Racosperma pycnanthum* (Benth.) Pedley, Austrobaileya 6 (3): 483 (2003).

**Type:** Australia: interior of New Holland (between the Loddon River and Pyramid Hill), *T. L. Mitchell 222* (?holotype: CGE). = Acacia petiolaris *Lehm.*, *in C.F.E. Otto, Neue Allg. Deutsche Gart.-Blumenzeitung 7: 210 (1851). Acacia westoni* Maiden, J. and Proc. Roy. Soc. New South Wales 54: 227 (1921).

**Distinctive features:** Shrub or tree 3–8 m high. Branchlets completely glabrous. Phyllodes often pendulous, falcately recurved to oblanceolate, 8–15 (−22.5) cm × (4–) 10–35 (–52) mm, obtuse to acute, coriaceous, glabrous, with prominent midrib, penninerved. Inflorescences racemose; raceme axes 2–9 cm long, stout, glabrous; heads showy, globular to obloid, 40–80-flowered, bright golden, sometimes lemon yellow. Pods linear, 5–13 cm × 5–7 (–8) mm, chartaceous, glabrous.

Native to Australia (Australian Capital Territory, New South Wales, South Australia, Victoria). It was introduced to Egypt in the early 1920s from Australia [44].

**Ecology and utilisation:** It is a crucial environmental weed in Western Australia, Tasmania and parts of New South Wales outside its native range. In South Africa, it competes with and replaces indigenous species. It is widely planted as an ornamental plant. The bark is one of the richest sources of tannin globally, although it is now rarely used commercially [70,71]. In Egypt, it had been cultivated in botanical gardens in the past.

**Selected specimens:** Giza, Cairo University Garden, 13.03.1973, *M. El Mahdi 5054* (CAI).

**17. *Acacia retinodes*** Schltdl., Linnaea 20 (6): 664 (1847); Delchev.Pl. Exo. Egypte: 4 (1871); Delchev. Prom. et jard. Caire: 63 (1899). ≡ *Racosperma retinodes* (Schltdl.) Pedley, Austrobaileya 6 (3): 484 (2003).

**Type:** Australia: Barossa valley, perhaps from Schlinckens Creek, Jan. 1844–1845, *H. H. Behr s.n.* (holotype: HAL-0041017! isotype: MEL-616152!).

**Distinctive features:** Tree up to 10 m high, occasionally suckering. Bark rough, furrowed, dark brown to black. Branchlets completely glabrous. Phyllodes variable, crowded on stems (4–10 mm apart), oblanceolate or linear, (5–) 6–16 cm × (2–) 3–12 (–16) mm, acuminate, uncinate, green to grey-green, glabrous, 1-veined per face, obscurely penninerved. Inflorescences racemose; raceme axes 2–4 (–5) cm long; heads globular, (16–) 18–30 (–34) −flowered, pale yellow to cream. Pods linear, up to 16 cm long, 8–11 mm wide, chartaceous.

Notes: According to [72], *Acacia retinodes* is a member of the ‘*Acacia microbotrya* group’ and is most closely related to *A. provincialis* and *A. uncifolia.* He used many morphological characters (habitat, habit, bark, phyllode characters, flower characters and pod width) to differentiate between the three species. In the present work, the phyllode characters and pod width are considered the most significant characters.

Native to Australia (South Australia). It was introduced to Egypt in the 19th century [41,43].

**Ecology and utilisation:** It is recorded as invasive according to the Global Invasive Species Database [52]. It is regarded as an environmental weed in Victoria and had become locally naturalised outside its native range [73,74] noted that *A. retinodes* has good prospects for future cultivation and development, for wood, tannin, fodder, seed and gum products. In Egypt, it is cultivated as an ornamental tree in historical and some private gardens.

**Selected specimens:** Giza, Maghraby Garden, s.d., *T. Labib s.n.* (Orman Herbarium).

**18. *Acacia salicina*** Lindl., in T.L. Mitchell, Three Exped. Australia 2: 20 (1838); Walsingham, Hort. Rev. 56: 66 (1922). ≡ *Racosperma salicinum* (Lindl.) Pedley, Austrobaileya 2 (4): 354 (1987).

**Type:** Australia: New Holland, [Lachlan R., 33°15′ S, 147°33′ E, N. S.W.], 30 Mar. 1836, *T. L. Mitchell 45* (CGE: holotype); Australia: Kingscote, Kangaroo Island, South Australia, [35°39′ S, 137°37′ E], January 1907, *J. H. Maiden s.n.* (syntype: NSW-212068!). *= Acacia varians* Benth., J. Exped. Trop. Australia 132 (1848).

**Distinctive features:** Shrub or tree 3–13 m high, often clonal due to suckering habit. Branchlets completely glabrous. Phyllodes pendulous, variable, linear to narrowly oblanceolate or narrowly elliptic, 7–20 cm × 4–30 mm, green to grey-green, glabrous, 1-veined, penninerved. Inflorescences 2–8-headed racemes; raceme axes 1–5 cm long, glabrous, rarely appressed-puberulous; heads globular, 15–25-flowered, cream to pale yellow. Pods narrowly oblong, up to 12 cm long, 7–13 mm wide, woody, thick, longitudinally striate when dry, grey-green, glabrous.

Native to Australia (New South Wales, Northern Territory, Queensland, South Australia, Victoria). It was introduced to Egypt in the early 1920s from Australia [44].

**Utilisation:** The pendulous habit and attractive foliage make it a desirable species for amenity planting. In Australia, sheep eat the leaves and pods. It has been planted as a fodder species in the arid zone of Libya and showed promise in semi-arid areas of Iran and Kuwait. The heartwood has been used for quality furniture. It is also used for shade, shelter, and ornamental purposes in North Africa and the Middle East. It suckers freely and can stabilize sandy areas and control erosion along stream banks [50]. In Egypt, it had been cultivated in botanical gardens in the past.

**Selected specimens:** Giza, Orman Garden, 12.11.1926, *M. Drar 4140* (CAIM).

**Figure 4:**
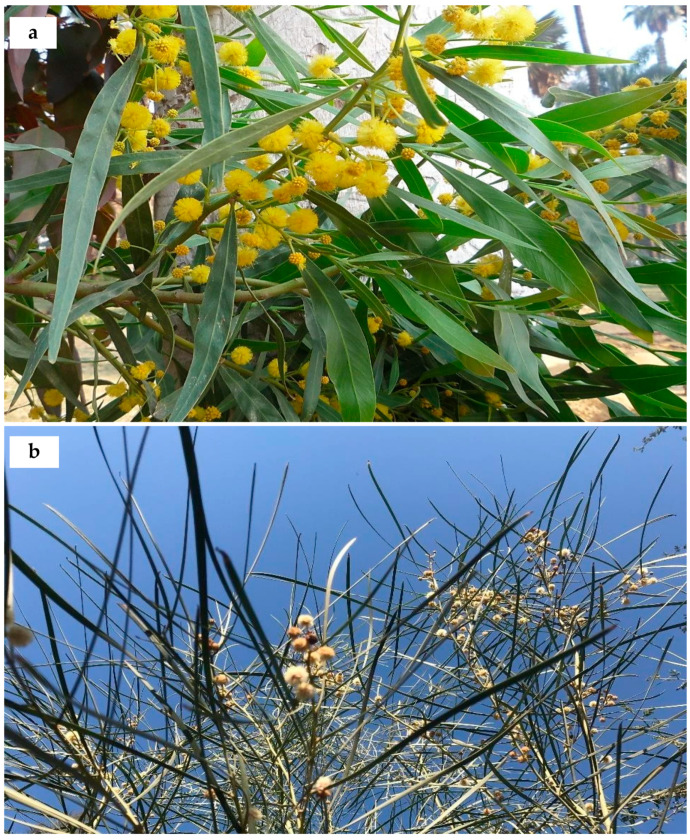
Examples of some studied *Acacia s.s.* species. (**a**) *Acacia saligna*, leaves reduced to phyllodes with golden yellow flowers in heads. (**b**) *Acacia calamifolia,* leaves reduced to phyllodes with pale yellow flowers in heads.

**19. *Acacia saligna*** (Labill.) H.L. Wendl., Comm. Acac. Aphyll. 4: 26 (1820); Delchev. Pl. Exo. Egypte: 4 (1871); Walsingham, Hort. Rev. 56: 66 (1922); Bircher, Gard. Hesperides: 340 (1960), [Figure 4a]. Basionym: *Mimosa saligna* Labill., Nov. Holl. Pl. 2: 86, pl. 235 (1807). ≡ *Racosperma salignum* (Labill.) Pedley, Austrobaileya 2: 355 (1987).

**Type:** Australia: Terra Diemen, [S. E. Tasmania] in error, *herb. Webbianum.* ex *herb. Labillardiere s.n.* (lectotype: FI-011176! left hand specimen on sheet, designated by B.R. Maslin, Nuytsia 1: 334, 1974)- Ibid loc., *Labillardiere s.n.* (isolectotype: P-02285050!). *Acacia cyanophylla* Lindl., Edward’s Bot. Reg. 25 (Misc.): 45 (1839); Delchev. Prom. et jard. Caire: 63 (1899); Bircher, Gard. Hesperides: 337 (1960). *Acacia lindleyi* Meisn., in J.G.C. Lehmann, Pl. Preiss. 1 (1): 14 (1844). *Acacia bracteata* Maiden and Blakely, J. Roy. Soc. Western Australia 13: 18, pl. 10, (1928).

**Distinctive features:** Shrub or tree (1–) 3–10 m high, often root-suckering. Bark grey, texture variable. Branchlets completely glabrous. Phyllodes patent to pendulous, linear to lanceolate, straight to falcate, 10–25 cm × 5–35 mm, green to glaucous, glabrous, with prominent midrib, finely penninerved. Inflorescences 2–10-headed racemes; raceme axes 2–60 mm long, glabrous; heads globular, 25–55-flowered, golden to lemon yellow. Pods linear, flat, shallowly constricted between seeds, 8–12 cm × 4–6 mm, coriaceous, glabrous.

Notes: It is a highly polymorphic species comprising four informal variants that have been assigned subspecies rank in some genetic studies [74,75,76]. It is currently under taxonomic review [77].

Native to Australia (Western Australia). It was introduced to Egypt in the 19th century [41,43], and listed among the seeds available for exchange [58].

**Ecology and utilisation:** It is a crucial environmental weed in the south-eastern parts of Australia and South Africa. It is recorded as invasive according to the Global Invasive Species Database [52]. It is a very adaptable species that can tolerate relatively dry, low nutrient soils and thrive in better conditions. It is planted widely for a range of purposes (e.g., windbreaks, sand dune fixation, fuelwoods, fodder production) in West Asia, South America, North and South Africa and parts of the Mediterranean [77,78]. In Egypt, it was cultivated as an ornamental tree in historical and many public gardens and streets. Recently, 1 million seedlings have been planted along the Mediterranean coast of Egypt for a range of rehabilitation [79]. It escaped from cultivation, spread invasively, and strongly affected biodiversity [80]. Currently, it is utilised in forest plantations in Gebel Elba natural forests, for sand dune fixation, as a bio-fertiliser and windbreaks. Tannin material extracted from the bark is used in tanning. Leaves, twigs, and fruits are used as fodder for livestock. Flowers are utilised in bee-keeping for honey production. Wood is used as firewood and in charcoal making [81].

**Selected specimens:** Giza, Orman Garden, 27.04.1963, *M. El Mahdi 5058* (CAI)- Qaliubiya, Qanatir, Barrage Medicinal Garden, 22.10.1968, *M. El Mahdi 5059* (CAI)- Alexandria: El Busseili sand dunes, 26.08.1952, *N. El Hadidi 5060* (CAI); Kharga Oasis, 13.03.1967, *N. El Hadidi* et al. *5061* (CAI).

**20. *Acacia vestita*** Ker Gawl., Bot. Reg. 9: pl. 698 (1823); Delchev. Prom. et jard. Caire: 64 (1899). ≡ *Racosperma vestitum* (Ker Gawl.) Pedley, Austrobaileya 6 (3): 492 (2003).

**Type:** Australia: New South Wales, *A. Cunningham* s.n. (?holotype: W-18890005129!) *= Acacia conspicua* Hort. ex Anon., Rev. Hort. 2: 31 (1835).

**Distinctive features:** Bushy shrub up to 4 m high. Branchlets hairy to some degree. Phyllodes inequilaterally ovate-elliptic, 10–20 × 4–10 mm, grey-green to ± glaucous, 1-veined per face. Inflorescences racemose; raceme axes 1.5–6 cm long, hirtellous; heads globular, 12–18-flowered, bright light golden. Pods narrowly oblong, rounded over seeds, up to 11 cm long, 10–14 mm wide, coriaceous, dark brown, glabrous, pruinose when young.

Native to Australia (New South Wales). It was introduced to Egypt in the 19th century [42].

**Utilisation:** It is widely cultivated as an ornamental species in Australia [82]. In Egypt, it is cultivated as an ornamental tree in khedivial and some private gardens.

**Selected specimens:** Giza, Maghraby Garden, 03.2008, *T. Labib s.n.* (Orman Herbarium).

**Group F:** Leaves reduced to phyllodes, bipinnate leaves found on seedlings, rarely persist; flowers arranged in globular heads; phyllodes with (1) 2 or more longitudinal nerves on at least one face.
1A. Phyllodes with 2 non-prominent longitudinal veins per face (1 when flat)**21. *A. calamifolia***1B. Phyllodes with more than 2 prominent longitudinal veins per face22A. Phyllodes reticulate between major longitudinal veins32B. Phyllodes not reticulate between major longitudinal veins**23. *A. harpophylla***3A. Phyllodes with inconspicuous secondary veins; racemes 2-headed**22. *A. cyclops***3B. Phyllodes with conspicuous secondary veins; racemes 3–5-headed**24. *A. melanoxylon***

**21. *Acacia calamifolia*** Sweet ex Lindl., Bot. Reg. 10: t. 839 (1824); Delchev. Prom. et jard. Caire: 63 (1899); Walsingham, Hort. Rev. 56: 66 (1922); Bircher, Gard. Hesperides: 337 (1960), Figure 4b. ≡ *Racosperma calamifolium* (Sweet ex Lindl.) Pedley, Austrobaileya 6 (3): 455 (2003).

**Type:** Australia: from the south-west interior of New Holland, Queensland, Mt Flinders [one of the peaks adjacent to L. Brewster which is c. 10 km S of Lachlan R. and c. 130 km N of Griffith, N.S.W.], June 1817, *A. Cunningham* 403 (isosyntype: K-000791537!). *= Acacia calamifolia* var. *pulverulenta* Domin, Biblioth. Bot. 89: 251 (1926).

**Distinctive features:** Shrub 2–4 m high. Phyllodes narrowly linear, terete to subterete, (2–) 2.5–9.5 (−10.5) cm × 1–1.5 mm, shortly acuminate with delicate, curved point, green to grey-green, glabrous, with 2 non-prominent longitudinal veins per face (1 when flat); veins not prominent and often ± impressed. Inflorescences 2–8 (–14)-headed racemes; raceme axes 10–25 (–30) mm long; heads globular to obloid, (28–) 30–44 (–46)-flowered, pale yellow to golden. Pods moniliform to submoniliform, up to 15 cm long, 3–6 mm wide, ± woody to crustaceous, wrinkled, glabrous.

Native to Australia (New South Wales, South Australia). It was introduced to Egypt in the 19th century [43].

**Utilisation:** It is planted for ornamental and amenity purposes in Australia. A potential source of seeds for human food [83]. It grows very well in the Egyptian environment and listed among the seeds available for exchange [58]. It is still cultivated in Mohamed Ali palace (Shubra) and some public gardens as an ornamental tree [40].

**Selected specimens:** Giza, Mazhar Botanic Garden, 15.01.2019, *R. Hamdy 5015* (CAI).

**22. *Acacia cyclops*** A. Cunn. ex G. Don, Gen. Hist. 2: 404 (1832), nom. cons.; Bircher, Gard. Hesperides: 337 (1960).

**Type:** Australia: Western Australia, King George’s Sound, *A. Cunningham 328* (syntype: K-000806233). *= Acacia mirbelii* Dehnh., Rivista Napol. 1: 168 (1839).

**Distinctive features:** Shrub to small tree 1–6 m high. Phyllodes ascending, narrowly oblong to elliptic or obovate, inequilateral, slightly recurved, 4–9.5 (–11) cm × (4–) 6–15 (–22) mm, obtuse or acute, apiculate, coriaceous, glabrous, with 3 or 4 distant main veins occasionally anatomosing with secondary veins. Inflorescences 2-headed racemes; raceme axes 3–20 mm long, somewhat compressed, glabrous or appressed-puberulous; heads globular, 5–7 mm diam., 60–75-flowered, golden. Pods linear, slightly raised over seeds, arcuate before dehiscence, up to 15 cm long, 7–15 mm wide, coriaceous, glabrous.

Native to Australia (South and Western Australia). It was introduced to Egypt in the 20th century [38].

**Ecology and utilization:** A significant environmental weed outside its native range within Australia and a potential threat in Victoria because of its invasiveness and significant environmental impacts elsewhere. It is also troublesome in South Africa, where it suppresses the indigenous vegetation and reduce species diversity. It is a drought-tolerant species and more tolerant to sea spray. Therefore, it is grown mainly to stabilise coastal sand dunes in North Africa (Tunisia, Libya, and Egypt). It also produces a dense, high-quality fuelwood [84,85]. In Egypt, it was cultivated as an ornamental tree in gardens.

**Selected specimens:** Alexandria, El Busseili Farm, 20.04.1950, *s.coll. Z7525* (CAIM)- Ibid Loc., 08.1952, *N. El Hadidi 5023* (CAI).

**23. *Acacia harpophylla*** F. Muell. ex Benth., Fl. Austral. 2: 389 (1864) ≡ *Racosperma harpophyllum* (F.Muell. ex Benth.) Pedley, Austrobaileya 2: 349 (1987).

**Type:** Australia: Queensland, Rockhampton, *Thozet s.n.* (holotype: K; isotype: MEL-2086557!).

**Distinctive features:** Tree up to 25 m high, root-suckering. Bark furrowed, almost black. Phyllodes falcate, 10–20 cm × 7–20 mm, coriaceous, sericeous, with numerous closely parallel veins of which 3–7 are more prominent than the rest. Inflorescences 2–8-headed racemes; raceme axes 2–10 mm long, appressed-puberulous; heads globular, 5–8 mm diam., 15–35-flowered, golden. Pods subterete, slightly constricted between seeds, straight to curved, up to 20 cm long, 5–10 mm wide, crustaceous, longitudinally veined, glabrous.

Native to Australia (New South Wales, Queensland). It was introduced to Egypt in the 20th century [45].

**Ecology and utilization:** It can form dense stands of root suckers when damaged, becoming a weed in some circumstances and is generally considered an undesirable agricultural and rangeland species. The timber has been used for firewood and fence posts [86]. In Egypt, it had been cultivated in botanical gardens in the past.

**Selected specimens:** Giza, Faculty of Agriculture, 26.10.1969, *M. Ezz El Din 5035* (CAI).

**24. *Acacia melanoxylon*** R.Br., in W.T. Aiton, Hortus Kew. ed. 2, 5: 462 (1813); Delchev. Pl. Exo. Egypte: 4 (1871); Delchev., Ap. Ge. végét. Exot. Égypte: 273 (1881); Asch. and Schweinf., Ill. Fl. Égypte, Mém. Inst. Égypt. 2: 72 (1887); Delchev. Prom. et jard. Caire: 63 (1899); Bircher, Gard. Hesperides: 339 (1960). ≡ *Racosperma melanoxylon* (R.Br.) Pedley, Bot. J. Linn. Soc. 92: 240 (1986).

**Type:** Australia, Derwent River, Tas., February–July 1804, *R. Brown*, sheet titled ‘Iter Australiense, 1802–5′ and bearing [Britten no.] *4364* (isolectotype: K-000806227! paratype: K-000806230! syntype: BM-000796848! isotype: E-00318362!) *= Acacia arcuata* Sieber ex Spreng., Syst. Veg., ed. 16, 3: 135 (1826). *Acacia melanoxylon* var. *obtusifolia* Ser., Fl. Jard. 3: 496 (1849).

**Distinctive features:** Tree 6–30 (–45) m high; sometimes a shrub 1.5–3 m high; may spread by root suckers. Bark fissured and scaly. Phyllodes narrowly elliptic, lanceolate or oblanceolate, inequilateral, straight to ± falcate, 4–16 cm × 6–30 mm, obtuse to acute, ± coriaceous, dark green, glabrous, with 3–5 main veins and prominently reticulate in between. Inflorescences 3–5-headed racemes; raceme axes 6–40 mm long; heads globular, 6 mm diam., 30–56-flowered, creamy pale yellow to white. Pods strongly curved to openly coiled and often twisted, up to 15 cm long, 3.5–8 mm wide, coriaceous to subwoody, glabrous.

Native to Australia (Australian Capital Territory, New South Wales, Queensland, South Australia, Tasmania, Victoria). It was introduced to Egypt in the 19th century [41,43].

**Ecology and utilisation:** It is mainly cultivated in Australia as an ornamental species in wetter areas. It has also been cultivated in forestry plantings in Hawaii, New Zealand, South Africa, Zimbabwe and Sri Lanka [87,88]. It is recorded as invasive according to the Global Invasive Species Database [52]. It has the potential to become naturalised in places where it is cultivated. In South Africa, the species has become an environmental weed difficult to control because of its fast growth rate, vigorous regrowth from root suckers and regeneration from seeds. In Egypt, it had been cultivated in historical and some public gardens in Cairo and Alexandria in the past [51]. According to [38], it is a valuable tree produces excellent timber used for furniture, railway carriages and building purposes.

**Selected specimens:** Giza, Faculty of Agriculture, 10.09.1968, *M. Ezz El Din* and *B. Diwan s.n.* (Orman Herbarium); El Saff, Alfred’s Bircher Garden, 17.05.1962, *V. Täckholm and I. El Sayed 5042* (CAI).

**B. *Senegalia*** Raf., Sylva Tellur., 119 (1838).

Lectotype: *Mimosa senegal* L. (vide Britton and Rose, N. Amer. Fl. 23: 106, 1928) (*Senegalia senegal* (L.) Britton [*Senegalia triacantha* Raf., nom. illeg.]).

Erect or climbing shrubs, lianas, or trees, with terete to angled branches. Stems striate, glabrous or with varied indumentum, armed with prickles. Stipules persistent or caducous. Leaves bipinnate, with sessile or stipitate extrafloral nectaries on the petiole or occasionally on the leaf and pinnae rachises. Inflorescences usually panicles, with flower-bearing axis capitate or spicate; floral bracts generally deciduous. Flowers whitish to yellowish, usually pentamerous, rarely tetramerous or hexamerous; calyx gamosepalous, glabrous or pilose; corolla gamopetalous, glabrous or pilose; nectarial disc present; stamens numerous, filaments free or fused at the base, anthers rimose, tipped by a caducous gland; ovary superior, stipitate, glabrous or pilose, stigma punctiform. Fruit legume, rarely follicle, flat, straight or falcate, chartaceous to coriaceous.

*Senegalia* occurs widely in the tropics and subtropics of both the Old and New Worlds [28], with its center of diversity in the neotropical region. According to WorldWide Wattle website [4], it has 217 species (two species in Australia, 56 in Asia, 62 in Africa, 97 in the Americas). In Egypt, only three native *Senegalia* taxa (two species, one subspecies) are present. In this study, 14 exotic taxa (eight species, three subspecies, three varieties) are recognized. They are accommodated in three informal groups (A–C).

Key to identify the studied *Senegalia* groups
1A. Prickles in pairs near the nodes**Group A**1B. Prickles in threes near the nodes**Group B**1C. Prickles scattered irregularly along the internodes (*Senegalia* sect. *Monacanthea*)**Group C**

**Group A:** Leaves bipinnate, stipules not spinescent, prickles in pairs near the nodes, flowers sessile or subsessile.
1A. Calyx glabrous21B. Calyx sparingly to densely puberulous or pubescent72A. Leaflets 1–5 pairs per pinna32B. Leaflets more than 5 pairs per pinna53A. Leaflets 1–2 pairs per pinna (rarely 3 or 4); trunk usually with knobby prickles**6. *S. nigrescens***3B. Leaflets 3–5 pairs per pinna; trunk usually without persistent prickles44A. Pods (5–) 8–18 × 2–3.5 cm; apex acuminate or apiculate**4. *S. goetzei* subsp. *goetzei***4B. Pods 5–7 × 0.8–1 cm; apex mucronate**5. *S. modesta***5A. Calyx 0.75–1.25 mm long, red or purplish65B. Calyx 1.5–3.5 mm long, normally not red or purplish**8. *S. polyacantha* subsp. *campylacantha***6A. Inflorescence 1.5–3 cm long; leaflets 11–17 pairs per pinna**7.*S. persiciflora***6B. Inflorescence (4–) 5–12 cm long; leaflets 12–45 pairs per pinna**3. *S. galpinii***7A. Leaflets 3–10 pairs per pinna**1. *S. burkei***7B. Leaflets 16–50 pairs per pinna**2. *S. catechu***

**1. *Senegalia burkei*** (Benth.) Kyal. and Boatwr., Bot. J. Linn. Soc. 172: 507 (2013).

Basionym: *Acacia burkei* Benth., London. J. Bot. 5: 98 (1846).

**Type:** South Africa: Transvaal, Macalisberg, *Burke* 126(holotype: K000244397; isotype: PRE- 0422920-0!).

**Distinctive features:** Tree 3–27 m high. Bark smooth or scaly, greyish-yellow to brownish or black. Stipules not spinescent. Prickles in pairs just below the nodes, brown when young, then grey to blackish, up to 3–8 mm long, strongly hooked downwards. Leaves bipinnate; rhachis ± densely pubescent, eglandular or with a gland between the top pair of pinnae only; pinnae (1–) 3–13 pairs; leaflets 3–10 pairs, obovate to oblong or elliptic, rounded to subacute or acute. Inflorescence spicate; spikes 3–6 (–14) cm long; flowers sessile or almost so, white; calyx 1.7–2.5 mm long, densely pubescent. Pods dehiscent, 4–17 × 1–2.5 cm, glabrous to hairy, linear-oblong, straight, purplish-brown, acuminate to mucronate at apex.

Native to Africa (Botswana, Mozambique, South Africa, Swaziland, Zimbabwe). It was introduced to Egypt in the 20th century [45].

**Ecology and utilisation:** It is found on sandy soils in hot and dry deciduous woodland. It secretes nectar, and its flowers attract many visitors. People, monkeys and bushbabies eat its gum. Bark and roots are used in traditional medicine to treat eye and back complaints. The wood is used to make furniture, tool handles and long-lasting fence posts. The heartwood makes a good quality fuel with coals that burn for a long time. Dry pods have a high nutritional value and are eaten by cattle [89]. In Egypt, it is cultivated as an ornamental tree in some private gardens.

**Selected specimens:** Giza, Private Garden, 14.11.2018, *R. Hassan 5013* (CAI); Mazhar Botanic Garden, 15.01.2019, *R. Hamdy 5014* (CAI).

**2. *Senegalia catechu*** (L.f.) P.J.H. Hurter and Mabb., Mabberley’s Pl.-Book, ed. 3: 1021 (2008).

Basionym: *Mimosa catechu* L.f., Suppl. Pl. ed.2: 439 (1782). ≡ *Acacia catechu* (L.f.) Willd., Sp. Pl., ed.4, 4(2): 1079 (1806); Delchev.Pl. Exo. Egypte: 3 (1871); Delchev., Ap.Ge. végét. Exot. Égypte: 272 (1881); Asch. and Schweinf., Ill. Fl. Égypte, Mém. Inst. Égypt. 2: 72 (1887); Walsingham, Hort. Rev. 56: 67 (1922).

**Type:** East Indies, s.d., *Willd. 73* (holotype: LINN-HS1598-36!). = *Acacia wallichiana* DC., Prodr. 2:458 (1825).

**Distinctive features:** Tree 6–10 m high. Bark greyish-brown, split into laminar strips. Stipules spiny, up to 8 mm long, hooked. Leaves bipinnate, with large gland near base of petiole; pinnae 12–30 pairs, with small glands between the pinnae; leaflets 16–50 pairs, linear, 2–6 × 1–1.5 mm, ciliate. Inflorescence spicate, axillary; spikes 2.5–10 cm long; flowers white or pale yellow; calyx campanulate, 1.2–1.5 cm, hairy. Pod brown, straight, strap-shaped, 12–15 × 1–2 cm, nitid, dehiscent, rostrate at apex.

Native to Indian Subcontinent (Bangladesh, Bhutan, India, Nepal, Pakistan); Southeast Asia (Myanmar). It was introduced to Egypt in the 19th century [41].

**Ecology and utilisation:** It is recorded as invasive according to the Global Invasive Species Database [52]. Extracts are used medicinally for a range of ailments. Tannins are used as a dye and for leather tanning. Wood for building construction and other purposes [26]. In Egypt, it had been cultivated in historical and some public gardens in Cairo and Alexandria [51].

**Selected specimens:** Giza, Faculty of Pharmacy Garden, Boulaq Dakrour, 15.10.1963, *M. El Mahdi 5017* (CAI)—Aswan: Botanic Island, 11.02.1961, *M. Abdallah 636* (CAIM).

**Figure 5:**
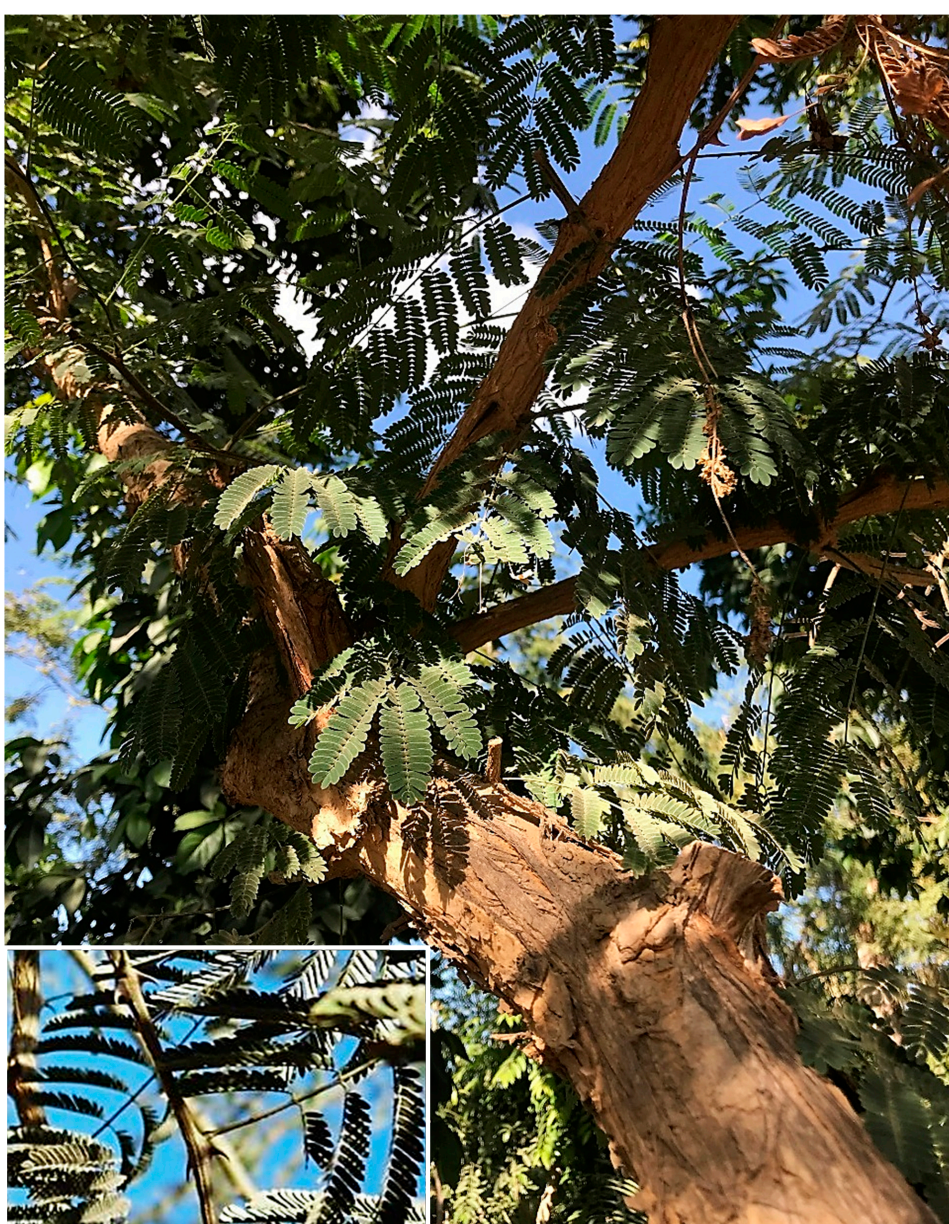
Examples of some studied *Senegalia* species. *Senegalia galpinii*, the corky rough bark, and bipinnate leaves with a pair of dark brown recurved prickles.

**3. *Senegalia galpinii*** (Burtt Davy) Seigler and Ebinger, Phytologia 92 (1): 93 (2010), (Figure 5).

Basionym: *Acacia galpinii* Burtt Davy, Bull. Misc. Inform. Kew 10: 326–327 (1922).

**Type:** South Africa: Transvaal, Waterberg District, Banks of Bad-zyn-loop River, Mosdene Estate, Naboomspruit, 19 September 1920, *E. E. Galpin 483M* (holotype: K; isotypes: GRA-0001172-0!, PRE-0423533-0!).

**Distinctive features:** Tree 8–25 m high. Bark rough, corky. Stipules not spinescent. Prickles in pairs just below the nodes, straight or recurved, up to 1 cm long. Leaves bipinnate; rhachis subglabrous to ± puberulous or pubescent, glandular between the top 1–4 pairs of pinnae; pinnae (4–) 9–14 pairs; leaflets 12–45 pairs, oblong to linear-oblong, obtuse to subacute. Inflorescence spicate; spikes (4–) 5–11 cm long; flowers sessile; calyx purple or reddish-purple, 0.75–1.25 mm long, glabrous. Pods purplish-brown, dehiscent, 11.5–28 × 2.7–3.5 cm, straight, glabrous.

Native to Africa (Botswana, Malawi, Mozambique, South Africa, Tanzania, Zambia, Zimbabwe). It was introduced to Egypt in the 20th century [45].

**Ecology and utilisation:** This tree can survive harsh conditions and makes a stunning tree along roads where there is enough space. Many insects such as bees and wasps visit the flowers. In the wild, the plant is grazed by different animals and used for shade during the hot summer [90]. In Egypt, it is cultivated as an ornamental tree in some private gardens.

**Selected specimens:** Giza, Private Garden, 14.11.2018, *R. Hassan 5032* (CAI); Mazhar Botanic Garden, 15.01.2019, *R. Hamdy 5033* (CAI).

**4. *Senegalia goetzei*** subsp. *goetzei* (Harms) Kyal. and Boatwr., Bot. J. Linn. Soc. 172 (4): 508 (2013).

Basionym: *Acacia goetzei* Brenan, Kew Bull. 11: 204 (1956).

**Type:** Tanzania: Kilosa District, Kidodi, *W. Goetze 387* (holotype: B); Burundi: Ruhembe, *W. Goetze 387* (isotype: E-00318381!).

**Distinctive features:** Tree 3–20 m high. Bark rough, grey or brown. Stipules not spinescent. Prickles in pairs just below the nodes, pale then dark-brown or grey, up to 7 mm long, hooked downwards. Pinnae 3–10 pairs; rhachis with a gland between the topmost pair of pinnae only; leaflets in 3–5 pairs, obovate, obovate-oblong or oblanceolate-oblong. Inflorescence spicate; spikes (2–) 3–12 cm long; flowers sessile, white or slightly yellowish; calyx glabrous. Pods dehiscent, (5–) 8–18 × 2–3.5 cm, glabrous, oblong or irregularly constricted, venose, red- to purplish-brown, acuminate or apiculate at apex.

Native to Africa (Angola, Democratic Republic of Congo, Kenya, Malawi, Mozambique, Somalia, Tanzania, Zambia, Zimbabwe). It was introduced to Egypt in the 20th century [45].

**Ecology and utilisation:** It is a nitrogen-fixing tree, growing on mixed dry woodland on moderately fertile shallow or stony soils [91]. In Egypt, it is cultivated as an ornamental tree in some private gardens.

**Selected specimens:** Giza, Mazhar Botanic Garden, 15.01.2019, *R. Hamdy 5034* (CAI).

**5. *Senegalia modesta*** (Wall.) P.J.H. Hurter, Mabberley’s Pl.-Book, ed. 3: 1021 (2008).

Basionym: *Acacia modesta* Wall., Pl. Asiat. Rar. 2: 27, pl. 130 (1831); Walsingham, Hort. Rev. 56: 67 (1922).

**Type:** India, cultivated in Calcutta Botanical Gardens, Saharanpur, 24 Apr.1825, *Wallich 5230 A and B* (syntypes: K-001120211!, K-001120212!, K-001120213!). *= Mimosa dumosa* Roxb., Hort. Bengal. 40 (1814). *Mimosa obovata* Roxb., Fl. Ind. Ed. 1832 2: 561 (1832).

**Distinctive features:** Tree 3–5 m high. Bark rough, brownish or greenish grey. Prickles in pairs, below the petiole, compressed, recurved, dark brown, 4–5 mm long, sometimes absent. Rachis 1–5 cm long, with a small gland near the base. Pinnae 2–3 pairs, 1–2.5 cm long; leaflets 3–5 pairs, 4–10 × 3–7 mm, ovate or obovate, oblique, obtuse, glaucous. Inflorescence spicate; spikes 3.5–7.5 cm long; calyx glabrous. Pod stipitate, dehiscing, 5–7 × 0.8–1 cm, thin, flat, straight, glabrous, deltoid mucronate at apex.

Native to Indian Subcontinent (India, Pakistan); Southeast Asia (Myanmar); West Asia (Afghanistan). It was introduced to Egypt in the early 1920s from East Indies [44].

**Ecology and utilisation:** It is a slow-growing tree, succeeds in dry and shallow soils. The established plants are very drought tolerant. Its gum is restorative, stimulant, tonic, and can treat muscular conditions, back pain, and stomach problems. The plant is used to treat dysentery, leprosy, oral toothache, trachoma, venereal diseases, and wounds. The tree is used as a pioneer for reforestation projects in arid and semi-arid locations. It is also grown to provide shelter from the wind [92]. In Egypt, it has occasionally been cultivated in botanical gardens and still growing in some gardens (e.g., Manial Palace).

**Selected specimens:** Qaliubiya, Barrage Medicinal Garden, 7.09.1959, *V. Täckholm 5044* (CAI)- Giza, Agricultural Museum Garden, 3.05.1947, *M. El Mahdi 5043* (CAI)-Cairo, Zohriya Garden, 17.11.1959, *V. Täckholm 5045* (CAI)-Aswan: Botanical Island, 15.04.1964, *M. El Mahdi 5046* (CAI).

**6. *Senegalia nigrescens*** (Oliv.) P.J.H. Hurter, Pl.-Book, ed. 3: 1021 (2008).

Basionym: *Acacia nigrescens* Oliv., Fl. Trop. Afr. 2: 340 (1871).

**Type:** Malawi: near Mitonda, Shire River, 19 Sept. 1854, *J. Kirk s.n.* (holotype: K-000244313). = *Acacia nigrescens* var. *pallens* Benth., Trans. Linn. Soc. London 30: 517 (1875). *Acacia perrotii* Warb., Notizbl. Königl. Bot. Gart. Berl. 2: 249 (1898). *Acacia passargei* Harms, in S. Passarge, Kalahari 789 (1904). *Acacia schliebenii* Harms, Notizbl. Bot. Gart. Berl.12: 507 (1935).

**Distinctive features:** Tree 3–30 m high; trunk with knobby prickles. Stipules not spinescent. Prickles in pairs just below nodes, hooked, blackish, 2.5–7 mm long. Pinnae 2–4 pairs; leaflets 1–2 pairs, (0.6−) 1–3.5 (–5) × (0.5−) 0.7–3 (–5) cm, obovate-orbicular to obovate-elliptic, subcoriaceous, emarginate at apex. Inflorescence spicate, aggregate or solitary; spikes 1–10 (–12) cm long; flowers white or cream; calyx glabrous. Pods dark brown, dehiscent, 6–18 × 1.5–2.5 cm, glabrous, oblong, hardly venose, acuminate at apex.

Native to Africa (Botswana, Malawi, Mozambique, Namibia, South Africa, Swaziland, Tanzania, Zambia, Zimbabwe). It was introduced to Egypt in the 2000s [45].

**Ecology and utilisation:** It is growing on woodland, bushland, wooded grassland, often near rivers and drainage lines; rarely on sandy soils [93]. The trees are the host of hole-nesting bird species. The wood is hard, drought- and termite-resistant but frost-tender, so it has been used to make fence posts and mine props. Leaves and pods are included in the diet of some animals [94]. In Egypt, it is cultivated as an ornamental tree in some private gardens.

**Selected specimens:** Giza: Private Garden, 14.11.2018, *R. Hassan 5048* (CAI); Mazhar Botanic Garden, 15.01.2019, *R. Hamdy 5049* (CAI).

**7. *Senegalia persiciflora*** (Pax) Kyal. and Boatwr., Bot. J. Linn. Soc. 172 (4): 509 (2013).

Basionym: *Acacia persiciflora* Pax, Bot. Jahrb. Syst. 39: 624 (1907).

**Type:** Ethiopia: Prov. Kutai, West-Shoa, Mumitscha-Urga Valley, 2200 m, 24 Mar. 1905 *Rosen s.n.* (holotype: WRSL). *= Acacia eggelingii* Baker f., J. Bot. 73: 263 (1935).

**Distinctive features:** Tree 4.5–9 (–15) m high. Bark scaling off in vertical strips, brownish-yellow. Stipules not spinescent. Prickles in pairs just below nodes, recurved, up to 3 mm long. Pinnae 4–8 pairs; rhachis pubescent, glandular between the top 1–5 pairs of pinnae; leaflets 11–17 pairs, 3–5.5 (–10) × 1–1.5(−2.5) mm, oblong-linear. Inflorescence spicate; spikes 1.5–3 cm long; flowers sessile or subsessile, precocious; calyx 0.75–1.25 mm long, red or purplish, glabrous. Pods brown, dehiscent, 6–15 × 1.5–2.5 cm, straight or slightly curved, venose.

Native to Africa (Democratic Republic of Congo, Ethiopia, Kenya, Sudan, Uganda). It was introduced to Egypt in the 20th century [45].

**Ecology and utilisation:** It is growing on woodland, wooded grassland and savanna on quartzite soil [95]. In Egypt, it had been cultivated in botanical gardens in the past.

**Selected specimens:** Qaliubiya, Barrage Medicinal Garden, 4.02.1968, *V. Täckholm 5052* (CAI).

**Figure 6:**
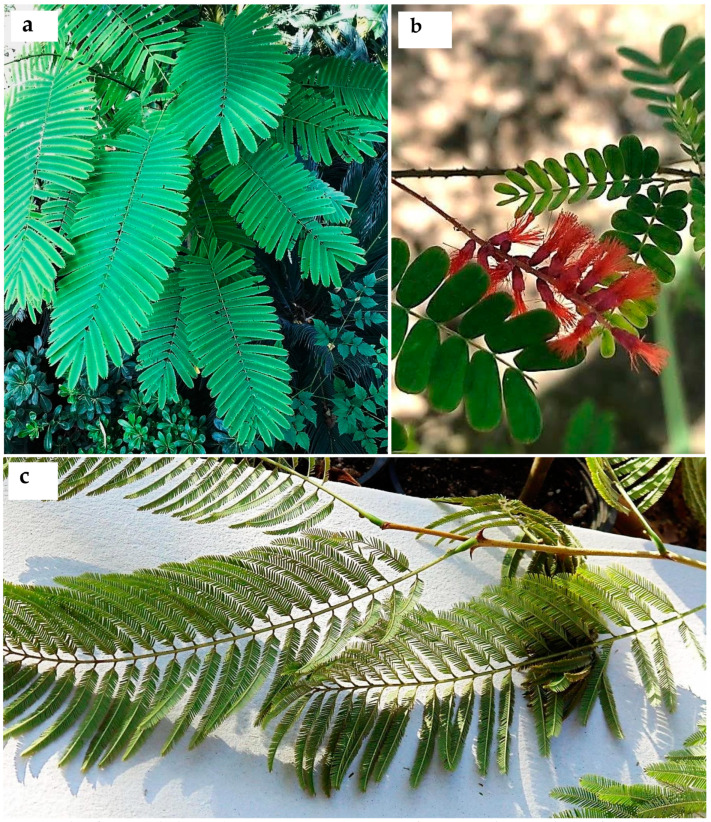
Examples of some studied *Senegalia* species. (**a**) *Senegalia pennata* subsp. *insuavis,* bipinnate leaves. (**b**) *Senegalia pervillei,* bipinnate leaves with characteristic red flowers in spikes. (**c**) *Senegalia polyacantha* subsp. *campylacantha,* bipinnate leaves with dark brown recurved prickles in pairs.

**8. *Senegalia polyacantha*** subsp. *campylacantha* (Hochst. ex A. Rich.) Kyal. and Boatwr., Bot. J. Linn. Soc. 172: 509 (2013), [Figure 6c].

Basionym: *Acacia campylacantha* Hochst. ex A. Rich., Tent. Fl. Abyss. 1: 242 (1847). ≡ *Acacia catechu* var. *campylacantha* (Hochst. ex A. Rich.) Roberty, Candollea 11: 157 (1948). *Acacia polyacantha* subsp. *campylacantha* (Hochst. ex A. Rich.) Brenan, Kew Bull. 11 (2): 195 (1956).

**Type:** Ethiopia: Prope Mai Dogale, 13 Nov. 1839, *Schimper 639* (syntype: P-00390998!); prope Dscheladscheranne ad latera montium versus fluvium Tacaze, 28 May 1840, *Schimper 893* (syntype: P-00390997!). = *Acacia erythrantha* Steud. ex A. Rich., Tent. Fl. Abyss. 1: 243 (1847).

**Distinctive features:** Tree 20–25 m high. Bark fissured, whitish to yellowish or grey. Stipules not spinescent. Prickles in pairs just below nodes, hooked, straw-coloured to brown or blackish, 4–12 mm long. Pinnae (6–) 13–40 (–60) pairs; leaflets (15–) 26–66 pairs, 2–5 (–6) × 0.5–0.75 (−1.25) mm, linear to linear-triangular. Inflorescence spicate; spikes (3.5−) 6–12.5 cm long; flowers cream or white; calyx 1.5–3.5 mm long, glabrous. Pods brown, dehiscent, 7–18 × 1–2 cm, oblong, straight, venose, glabrous or rarely pubescent, acuminate at the apex.

Native to Africa (Benin, Botswana, Burundi, Cameroon, Central African Republic, Ethiopia, Gambia, Ghana, Ivory Coast, Kenya, Malawi, Mali, Mozambique, Niger, Nigeria, Rwanda, Senegal, South Africa, Sudan, Tanzania, Togo, Uganda, Zambia, Zimbabwe). It was introduced to Egypt in the 2000s [45].

**Ecology and utilisation:** It is a very fast-growing tree, short-lived (30–40 years), sensitive to cold and frost, growing on wooded grassland, deciduous woodland and bushland, and open woodland with good grass cover [96]. The gum is used in confectionery products and as an adhesive, while the bark is used in tanning. The roots and bark are used for medicinal and magical purposes [97]. In Egypt, it is cultivated as an ornamental tree in some gardens. It grew well in botanical gardens in the past, under the name *Acacia suma*, but unfortunately, no specimens are left to identify the subspecies [38,44].

**Selected specimens:** Giza, Zoological Garden, 07.1952, *L. Boulos 5016* (CAI).

**Group B:** Leaves bipinnate, stipules not spinescent, prickles in threes near the nodes, the two laterals usually curved upwards, and the central one downwards.
-Pod apex rounded to acute, seldom acuminate**9. *S. senegal* var. *senegal***-Pod apex usually strongly acuminate or rostrate**10. *S. senegal* var. *rostrate***

**9. *Senegalia senegal*** var. *senegal* (L.) Britton in Britton and P. Wilson, Sci. Surv. Porto Rico and Virgin Islands 6: 538 (1930).

Basionym: *Mimosa senegal* L., Sp. Pl.1: 521 (1753); Delile, Descr. Égypte, Hist. Nat. 2: 79 (1813); ibid. 19: 111 (1824). *≡ Acacia senegal* (L.) Willd., Sp. l. 4(2): 1077(1806)*;* Asch. and Schweinf., Ill. Fl. Égypte, Mém. Inst. Égypt. 2: 72 (1887); Walsingham, Hort. Rev. 56: 67 (1922); Bircher, Gard. Hesperides: 340 (1960).

**Type:** Senegal, *Herb.* *Adanson 16899* (neotype: P, designated by Ross, 1979).

**Distinctive features:** Shrub or tree up to 13 m high. Bark scaly, rough, grey to brown or blackish. Stipules not spinescent. Prickles usually in threes, the central one hooked downwards, the laterals curved upwards, up to 7 mm long. Pinnae (2–) 3–6 (–12) pairs, 0.5–1.5 (−2.5) cm long; leaflets 7–25 pairs, 1–4 (–9) × 0.5–2 (–3) mm, linear-to elliptic-oblong. Inflorescence spicate; spikes 1.5–10 cm long; flowers white or cream, fragrant. Pods grey-brown, dehiscent, (2–) 4–19 × (1–) 2–3.5 cm, densely to sparsely appressed-pubescent to puberulous, oblong, straight, venose, rounded to acute at apex.

Native to Africa (Cameroon, Ethiopia, Kenya, Mozambique, Nigeria, Senegal, Somalia); Indian Subcontinent (India). It was introduced to Egypt in the 19th century [41,43].

**Ecology and utilisation:** It is growing on wooded grassland, bushland, sand dunes and sandy Sahelian soils [98]. In Egypt, it had been cultivated in historical and some public gardens in Cairo and Alexandria (Ascherson and Schweinfurth, 1887). It was listed among the seeds available for exchange [58] and is still grown in some parts of Egypt for its gum [99].

**Selected specimens:** Giza, in front of the Agricultural Museum, 30.08.1947, *M. Drar 5061* (CAI).

**10. *Senegalia senegal*** var. *rostrata* (Brenan) Kyal. and Boatwr., Bot. J. Linn. Soc. 172: 510 (2013).

Basionym: *Acacia senegal* var. *rostrata* Brenan, Kew Bull. 8 (1): 99–100 (1953).

**Type:** South Africa: Transvaal, Soutpansberg District, Dongola Reserve, *C. Verdoorn 2264* (holotype: K-000244368!; isotype: PRE-0423574-0!). *= Acacia oxyosprion* Chiov. var. *oxyosprion*, Fl. Somala 2: 188 (1932).

**Distinctive feature:** Pod apex usually strongly acuminate or rostrate.

Native to Africa: Angola, Botswana, Kenya, Mozambique, Namibia, Somalia, South Africa, Swaziland, Uganda, Zimbabwe. It was introduced to Egypt in the 20th Century [45].

**Utilisation:** In Egypt, it is cultivated as an ornamental tree in some private gardens.

**Selected specimens:** Giza, Private Garden, 14.11.2018, *R. Hassan 5063* (CAI).

**Group C (*Senegalia* sect. *Monacanthea*):** Leaves bipinnate, stipules not spinescent, prickles scattered irregularly along the internodes.
1A. Inflorescence spicate21B. Inflorescence capitate**13. *S. pennata* subsp. *insuavis***2A. Flowers creamy-white in colour32B. Flowers red in colour**14. *S. pervillei* var. *pervillei***3A. Pods deep red to purplish red, semi-translucent, straight**11. *S. ataxacantha***3B. Pods light brown, papery, twisted**12. *S. greggii***

**11. *Senegalia ataxacantha*** (DC.) Kyal. and Boatwr., Bot. J. Linn. Soc. 172: 507 (2013).

Basionym: *Acacia ataxacantha* DC., Prodr. 2: 459 (1825).

**Type:** Senegal, s.d., *Bacle s.n.* and *Perrottet s.n.* (syntypes: G-DC). *= Acacia eriadenia* Benth. in Hook., Lond. J. Bot. 5: 98–99 (1846). Albizia mossambicensis Bolle, in W. Peters, Naturw. Reise Mossambique 6(1): 4 (1861). *Acacia lugardiae* N.E.Br., Bull. Misc. Inform. Kew Bull.: 107 (1909). *Acacia caffra* var. rupestris Sim, Forest Fl. Port. E. Afr. 56, pl. 39b (1909).

**Distinctive features:** Scandent shrub up to 15 m high or a straggling non-climbing shrub or small tree 2–10 m high. Stipules not spinescent. Prickles scattered along the internodes, hooked or deflexed, broad-based, up to 7 (–15) mm long. Pinnae (4–) 6–25 (–29) pairs; rhachis 5–13 cm long, prickly or unarmed; leaflets 14–62 pairs, 2–5 (−7.5) × 0.5–1.5 mm. Inflorescence spicate; spikes 4–10 cm long; flowers cream to white. Pods deep red to purplish red, semi-translucent, straight, dehiscent, 5–20 × 1–2.5 cm, linear-oblong, acuminate at both ends or sometimes merely subacute at the apex, puberulous or almost glabrous.

Native to Africa (Angola, Benin, Botswana, Cameroon, Central African Republic, Chad, Guinea-Bissau, Ivory Coast, Kenya, Liberia, Mali, Mozambique, Namibia, Niger, Nigeria, Senegal, Sierra Leone, South Africa, Sudan, Swaziland, Tanzania, Togo, Zimbabwe). It was introduced to Egypt in the 2000s [45].

**Ecology and utilisation:** It grows in drier areas, woodlands, wooded grassland, sometimes in Lowveld in riverine areas forming thickets [100]. It is resistant to decay due to gum deposits. The wood can be split into paper-like strips without cracking, and these strips are commonly used as weaving material for baskets. The roots are also used in basketry and in making long-stem tobacco pipes. It is used in traditional medicine to treat constipation and abdominal pains [101]. In Egypt, it is cultivated as an ornamental tree in some private gardens.

**Selected specimens:** Giza, Private Garden, 14.11.2018, *R. Hassan 5008* (CAI); Mazhar Botanic Garden, 15.01.2019, *R. Hamdy 5009* (CAI).

**12. *Senegalia greggii*** (A. Gray) Britton and Rose, N. Amer. Fl. 23 (2): 110 (1928).

Basionym: *Acacia greggii* A. Gray, Pl. Wright. 1: 65 (1852); Bircher, Gard. Hesperides: 338 (1960).

**Type:** Mexico, Dry valley west of Patos, 10 Apr. 1847, *J. Gregg s.n.* (lectotype: GH-00058205! designated by Isley, 1969). = *Acacia durandiana* Buckley, Proc. Acad. Nat. Sci. Philadelphia 1861: 453 (1862). *Acacia greggii* var. *arizonica* Isely, Sida 3: 377 (1969).

**Distinctive features:** Shrub or tree 10–15 m high. Pinnae 1–3 pairs; leaflets 3–7 pairs, obovate to narrowly oblong, pale, obtuse at apex, 4–6 mm long. Inflorescence raceme, spicate; spikes 2–2.5 cm long. Pods light brown, 5–7.5 × 1.5 cm, papery, twisted, glabrous or tomentose, rounded at apex.

Native to Central America (Mexico); North America (Arizona, California, Nevada, New Mexico, Texas, Utah). It was introduced to Egypt in the 20th century [38].

**Utilisation:** It is one of the most common and iconic desert scrub plants where found. Its flowers are one of the most important nectar sources for honey bees [102]. In Egypt, it had been cultivated in botanical gardens in the past.

**Selected specimens:** Giza, Agricultural Museum Garden, 30.08.1956, *M. El Mahdi 0017026* (CAIM).

**13. *Senegalia pennata*** subsp. *insuavis* (Lace) Maslin, Seigler and Ebinger, Blumea 58: 42 (2013), [Figure 6a].

Basionym: *Acacia insuavis* Lace, Bull. Misc. Inform. Kew 1915: 401 (1915). ≡ *Acacia pennata* subsp. *insuavis* (Lace) I.C. Nielsen, Adansonia, ser. 2, 19: 353 (1980). *Senegalia insuavis* (Lace) Pedley, Austrobaileya 9(2): 314 (2014).

**Type:** Myanmar, Maymyo Plateau, near Ani Sakan, alt. 3000 ft., 18 May 1913, *J.H. Lace 6173* (lectotype: E-00318280, designated by Maslin et al., 2019).

**Distinctive features:** Lianas or shrubs up to 5 m high. Pinnae 17–28 pairs, 3–10 cm long; leaflets 4–6 × 0.5–1 mm. Stipules caducous. Prickles few to numerous on internodes, straight to recurved. Inflorescences capitate; heads arranged in terminal, elongated racemes or open panicles, white or creamy white to pale yellow, (30–) 35–55-flowered. Pods oblong, 12–23 × (1.5−) 2–2.5 (–3) cm, chartaceous to coriaceous, straight to very shallowly curved, glabrous.

Notes: According to [26], the disagreeable odour that is emitting from fresh leaves or branchlets when crushed, and leaflets that often obviously curved forward and/or folded lengthwise when dry are the most distinctive features for this subspecies in this study.

Native to East Asia (China); Southeast Asia (Myanmar). It was introduced to Egypt in the 20th century [45].

**Utilisation:** The soft new shoots are commonly used in Asian cooking and as a hedge-row shrub in Thailand [26]. According to [103], the roots of this subspecies in Laos are used in local medicine to combat anemia. In Egypt, it is cultivated as an ornamental tree in some private gardens.

**Selected specimens:** Giza: Private Garden, 14.11.2018, *R. Hassan 5050* (CAI); Mazhar Botanic Garden, 15.01.2019, *R. Hamdy 5051* (CAI).

**14. *Senegalia pervillei*** var. *pervillei* (Benth.) Boatwr., Bot. J. Linn. Soc. 179 (2): 292 (2015), [Figure 6b].

Autonym: *Acacia pervillei* var. *pervillei* Benth., Legum. Madagascar 237 (2002).

**Type:** Madagascar: Mahajanga, Melaky, Ambongo, Grimpant sur les grands arbres, fleurs rouges superbes, 15 Feb. 1841, *A. Pervillé 613* (holotype: P).

**Distinctive features:** Scrambling, prickly shrub or liana 1–12 m high. Stipules deciduous. Prickles numerous on internodes, slightly recurved, glabrous. Leaf rhachis puberulent, (2–) 5.5–11 cm long, with a gland between the apical pair of pinnae; pinnae 3–10 pairs; leaflets 8–20 (–26) pairs per pinna, deep green above pale below, 4–15 × (1–) 2–5 mm, oblong to narrowly oblong. Inflorescence spicate, dense, 2–6 cm long; flowers crimson-red. Pods pale brown, 11–13 × 2 cm, oblong, flat, with acute apex.

Endemic to Madagascar. It was introduced to Egypt in the 2000s [45].

**Utilisation:** In Madagascar, powder from grated stems is rubbed around forehead to cure headache. Leaf tisanes are taken to cure diarrhoea [104]. In Egypt, it is cultivated as an ornamental tree in some private gardens.

**Selected specimens:** Giza, Mazhar Botanic Garden, 15.01.2019, *T. Labib s.n.* (MAZHAR).

**C. *Vachellia*** Wight and Arn., Prodr. Fl. Ind. Orient., 1: 272 (1834).

Type species: Vachellia farnesiana (L.) Wight and Arn., Prodr. Fl. Ind. Orient. 1: 272 (1834).

Trees or shrubs, sometimes climbing, always armed with spiny stipules, situated near the leaf bases. Leaves alternate, bipinnate, with opposite pinnae. Inflorescences racemose; flowers bisexual, rarely unisexual, yellow or creamy white, in spherical heads, or rarely elongated spikes; calyx and corolla 4 to 5-lobed; stamens numerous. Glands usually present on the rachis and the upper side of the petiole. Pods straight, curved or curled, dehiscent or indehiscent.

*Vachellia* is a wide-ranging genus, has a pantropical distribution. According to WorldWide Wattle website [4], it has 164 species (nine species in Australia, 33 in Asia, 72 in Africa, 61 in the Americas). In Egypt, only 11 native taxa (two species, six subspecies, three varieties) are recorded. In this study, 13 exotic taxa (eight species, one subspecies, four varieties) are recognized. They are accommodated in three informal groups (A–C).

Key to identify the studied *Vachellia* groups:
1A. Flowers in round heads21B. Flowers in cylindrical spikes**Group C**2A. Flowers bright golden to orange-yellow**Group A**2B. Flowers yellowish-white, cream, white, rarely pink**Group B**

**Group A:** Leaves bipinnate; stipules spinescent, in pairs; flowers in round heads, bright golden to orange-yellow.
1A. Involucel apical; leaflets not spinulose-mucronate apically 21B. Involucel basal or in the middle; leaflets spinulose-mucronate apically52A. Petiolar gland absent; spines stout, usually fused basally and swollen into enlarged “ant galls”**3. *V. erioloba***2B. Petiolar gland present; spines slender, never forming “ant galls”33A. Mature pods dark brown to black, not constricted between the seeds43B. Mature pods light brown, strongly constricted between the seeds**2. *V. constricta***4A. Midvein and lateral veins obvious and slightly raised; pods subterete to terete, obliquely to longitudinally finely striate**4. *V. farnesiana* var. *farnesiana***4B. Midvein and lateral veins not obvious; pods oblong-elongate or subglobose, usually not striate**1. *V. caven***5A. Some spines fused basally into inflated ± bilobed “ant galls” or the spines fusiform inflated65B. Spines not inflated into ± bilobed “ant galls” or fusiform inflated**7. *V. xanthophloea***6A. “Ant galls” composed of pairs of spines fused below into an inflated ± bilobed structure**6. *V. seyal* var. *fistula***6B. “Ant galls” composed of pairs of spines, each spine fusiform-inflated but not fused with the other member of the pair except basally and free for almost its entire length**5. *V. karroo***

**1. *Vachellia caven*** (Molina) Seigler and Ebinger, Phytologia 87 (3): 148 (2005).

**Type:** Chile: Raneagna, Oct. 1828, *C.G. Bertero s.n*. (lectotype: SGO, designated by Aronson, 1992). = *Mimosa caven* Molina, Sag. Stor. Nat. Chili, ed. 1: 174, 355 (1782). *Acacia caven* Molina, Sag. Stor. Nat. Chili, ed. 2: 163–164, 299 (1810). *Acacia cavenia* (Molina) Hook. and Arn. Bot. Beechey Voy.: 21 (1841); Delchev. Pl. Exo. Egypte: 3 (1871); Delchev., Ap. Ge. végét. Exot. Égypte: 273 (1881); Asch. and Schweinf. Ill. Fl. Égypte, Mém. Inst. Égypt. 2: 72 (1887); Bircher, Gard. Hesperides: 337 (1960). *Acacia farnesiana* var. *cavenia* (Hook and Arn.) O. Kuntze, Rev. Gen. P1. 3:47 (1898).

**Distinctive features:** Shrub or tree up to 10 m high. Bark dark-grey to brown. Stipules spinescent, up to 1.5 cm long, slender. Petiolar gland present. Leaf rhachis 0.5− 4.5 cm long, a small gland usually at the junction of the top 1–3 pinna pairs. Pinnae 3–10 pairs, 1.2–2.2 cm long; leaflets 11–26 pairs per pinna, 1–4 × 0.4–0.9 mm, oblong. Inflorescence capitate, solitary or in fascicles; flowers bright yellow; involucel apical. Pods dark-brown to black, 2–9.5 × 1.3–2.5 cm, indehiscent, oblong-elongate or subglobose, straight, terete, apex narrowing to a short beak up to 10 mm long, glabrous.

Native to South America (Argentina, Bolivia, Chile, Paraguay, and Uruguay). It was introduced to Egypt in the 19th century [41,43].

**Ecology and utilisation:** A fast-growing plant succeeds in warm temperate to subtropical and tropical areas, grows well in slightly acidic to acidic soil with a significant content of organic matter. Drought tolerant but cannot withstand temperatures below freezing. It can be used as a hedge and in planting programs for restoring native woodland. The seeds can be toasted and used as a coffee replacement. The cooked bark can heal sores and wounds [105]. In Egypt, it had been cultivated in historical gardens in the past.

**Selected specimens:** Cairo, Zohriya Garden, 17.11.1959, *V. Täckholm 5019* (CAI).

**2. *Vachellia constricta*** (Benth.) Seigler and Ebinger, Phytologia 87 (3): 152 (2005).

**Type:** USA: Mexico, Chihuahua, May-Oct. 1849, *C. Wright 162* (holotype: K-000117588!). = *Acacia constricta* var. *paucispina* Wooton and Standl., Bull. Torrey Bot. Club 36 (2): 105–106 (1909); Walsingham, Hort. Rev. 56: 67 (1922).

**Distinctive features:** Shrub or tree up to 16 m high. Bark slightly rough, light grey to mahogany colored. Stipules spinescent, 0.3–0.6 (–2) cm long, slender. Petiolar gland present, a small gland usually at the junction of the terminal pairs of pinnae. Pinnae 3–6 pairs, 2.5–4 cm long; leaflets 5–9 (–16) pairs per pinna, 2–3.5 × 0.7–1.3 mm. Inflorescence capitate, solitary or in fascicles; flowers yellow, fragrant; involucel apical. Pods light brown, (3–) 7–14 × 1–2.3 cm, dehiscent, straight to slightly curved, glabrous, strongly constricted between the seeds.

Native to Central America (Mexico); North America (Arizona, New Mexico, Texas). It was introduced to Egypt in the 20th century from Mexico [44].

**Ecology and utilisation:** It is an attractive shrub with fragrant, showy orange-yellow flowers that attract nectar-seeking insects, butterflies and bees. It is well adapted to the arid conditions of desert life, and prefers dry slopes, washes, flat desert areas and mesas. Livestock will consume the fruit pods but do not appear to relish the foliage [106]. In Egypt, it had been cultivated in gardens (e.g., Orman garden) as an ornamental shrub in the past.

**Selected specimens:** Giza, Orman Botanical Garden, 25.08.1969, *M. El Mahdi 5021* (CAI).

**3. *Vachellia erioloba*** (E. Mey.) P.J.H.Hurter, Mabberley’s Pl.-Book, ed. 3: 1021 (2008).

Basionym: *Acacia erioloba* E. Mey., Comm. Pl. Afr. Austr.1: 171 (1836), non Edgew (1847).

**Type:** South Africa: Transvaal: Wolmaransstad District, between Kommandodrif and Makwassie, 22 Jan. 1968, *J. W. Morris 1042* (neotype: K-000244430!, designated by Ross, 1979). = *Acacia giraffae sensu auct. mult.,* non *A. giraffae* Willd. (1809) *sensu stricto*; Burch., Trav. Int. S. Afr. 2: 240 (1824); Bircher, Gard. Hesperides: 338 (1960). *Acacia giraffae* var. *espinosa* Kuntze, Jahrb. K. Bot. Gart. Mus. Berl. 4: 264 (1886).

**Distinctive features:** Shrub or tree up to 22 m high. Bark rough with deep longitudinal fissures, grey, greyish-brown to blackish. Stipules spinescent, 0.5–5 (–10) cm long, stout, often fused together basally and swollen into enlarged ant-galls. Petiolar gland present. Leaf rhachis (0–) 1–5.5 cm long, a small gland usually at the junction of each pinna pair. Pinnae (1–) 2–5 pairs, 1.3–4.2 cm long; leaflets (6–) 8–18 pairs per pinna, 4–13 × (0.7−) 1.5–4.5 mm. Inflorescence capitate, solitary or in fascicles; flowers bright golden-yellow, fragrant; involucel apical. Pods greyish-green, (4–) 6–13 × 1.8–5 cm, indehiscent, semi-lunate to suborbicular, densely grey-velutinous.

Native to Africa (Angola, Botswana, Namibia, South Africa, Zambia, Zimbabwe). It was introduced to Egypt in the 20th century from South Africa under the name “camel thorn” [38].

**Ecology and utilisation:** It is a very highly invasive species in Australia that can displace preferred vegetation. It is a long-lived plant that grows in sandy areas and can tolerate hot summer temperatures and severe frosts. The pods are useful fodder for cattle and wild animals in Africa. Dry powdered pods can be used to treat ear infections, gum to treat gonorrhea, burned bark to treat headaches and root to treat toothache [107]. In Egypt, the wood was used as fuel [38]. It is cultivated as an ornamental tree in some gardens [45].

**Selected specimens:** Giza, Mazhar Botanic Garden, 15.01.2019, *R. Hamdy 5025* (CAI).

**4.*Vachellia farnesiana*** var. *farnesiana* (L.) Wright and Arn., Phytologia 87 (3): 157 (2005). Autonym: *Acacia farnesiana* (L.) Willd. var. *farnesiana* = *Acacia acicularis* Humb. and Bonpl. ex Willd., Enum. Pl. 1056 (1809).

**Type:** Aldinus: Exactissima descriptio rariorum plantarum Romae in Horto Farnesiano: 2–7 (1625), which provides, under the name Acacia Indica Farnesiana, a detailed description and two illustrations of a plant in cultivation in the garden of Cardinal Farnese in Rome (lectotype, designated by Ross, 1975).

**Distinctive features:** Shrub or tree up to 4 m high. Bark smooth or finely fissured, dark grey to brown. Stipules spinescent, 0.5–1.5 (–3) cm long, slender. Petiolar gland present. Leaf rhachis 2–5 cm long, a small gland below the junction of the top pinna pair. Pinnae 2–7 pairs, 0.7–4.2 (–5) cm long; leaflets 10–21 pairs per pinna, 3–6 × 0.5–1.8 mm, mid vein and lateral veins obvious and slightly raised. Inflorescence capitate, solitary or in fascicles; flowers bright yellow, fragrant; involucel apical. Pods dark brown to blackish, 4–7.5 × 1–1.5 (–2) cm, tardily dehiscent, oblong, straight or falcate, subterete to terete, obliquely and longitudinally finely striate, glabrous.

Native to Central America (Belize, Guatemala, Honduras, Mexico; North America (California, Florida, Louisiana, Texas). It was introduced to Egypt a long time ago, probably from tropical America [108], then cultivated and become naturalized.

**Ecology and utilisation:** It is recorded as invasive according to the Global Invasive Species Database [52]. The foliage and green pods are palatable to cattle and sheep. It is favoured for its fragrant flowers and cultivated in Mediterranean countries for its essential oil to manufacture perfumes. Although the growth rate is slow, it is valuable ornamentally and used as hedges around citrus groves, especially in Egypt and Israel [109,110]. Its flowers produce a perfume called “cassie” extensively used in European perfumery. In Egypt, the bark and pods are used for dying and tanning leather [111], and the wood is mainly used for fuel [38].

**Selected specimens:** Qaliubiya, Qanatir, Barrage Medicinal Garden, 8.01. 1961, *V. Täckholm 5027* (CAI)- Giza, 28.09.1910, *E. Hartmann 5026* (CAI); El Saff, Alfred Bircher’s Garden, 24.05.1962, *V. Täckholm* and *I. El Sayed 5028* (CAI); Orman Garden, 15.03.2016, *S. Abd El Zaher s.n.* (Orman Herbarium)- Aswan, Aswan Botanic Island, 24.03.1999, *H. Rofaeel 29395* (CAIM).

**Figure 7:**
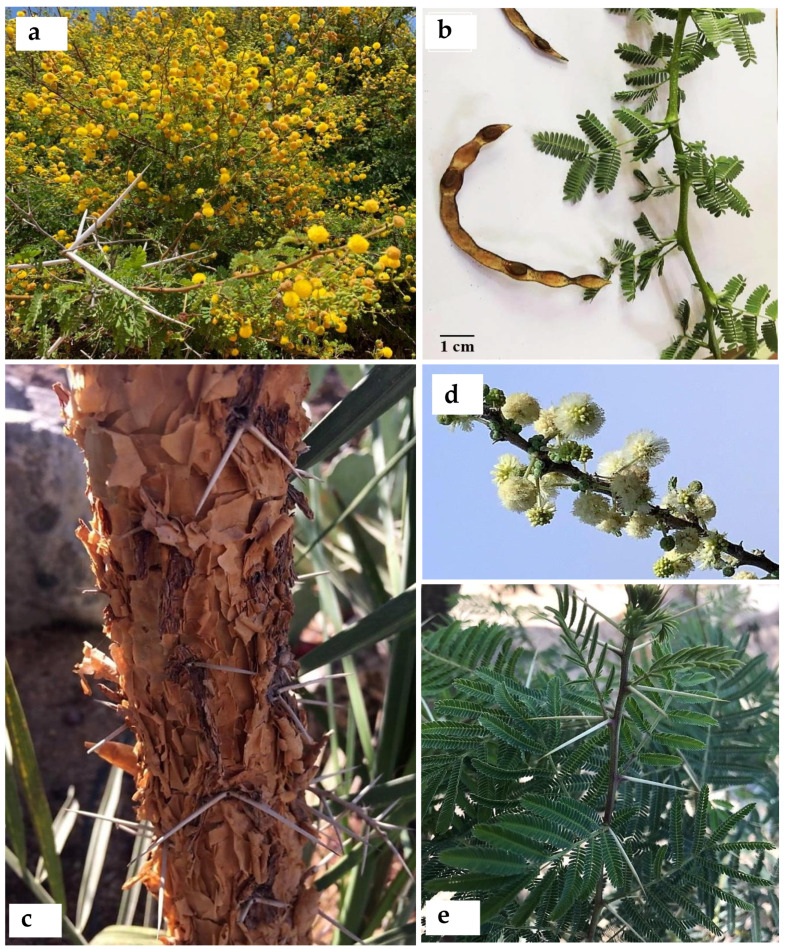
Examples of some studied *Vachellia* species. (**a**,**b**) *Vachellia karroo*, (**a**) golden-yellow flower heads and distinctive long white thorns in pairs, (**b**) bipinnate leaves with brown constricted pod. (**c**–**e**) *Vachellia sieberiana* var. *woodii,* (**c**) papery bark with long white thorns, (**d**) creamy-white flower heads at anthesis, (**e**) bipinnate leaves with long white thorns in pairs.

**5. *Vachellia karroo*** (Hayne) Banfi and Galasso, Atti. Soc. Ital. Sci. Nat. Mus. Civico Storia Nat. Milano 149: 149 (2007), [Figure 7a,b].

Basionym: *Acacia karroo* Hayne, Getreue Darstell. Gew., 10, pl. 33 (1827).

**Type:** South Africa: Cape Province, locality unknown, 9 Apr. 1944, Herb. *Willdenow 19184*, fol. 2 (lectotype: B; isotype: PRE, designated by Kyalangalilwa et al., 2013). = *Mimosa leucacantha* Jacq., Pl. Hort. Shoenbr. 3: 75, pl. 393 (1798). *Acacia horrida* (L.) Willd., Sp. Pl., ed. 4, 4(2): 1082 (1806), pro parte. *Acacia hirtella* E. Mey., Comm. Pl. Afr. Austr. 1: 167 (1836).

**Distinctive features:** Shrub or tree up to 12 (22) m high. Bark rough, longitudinally fissured, reddish-brown or dark brown or black. Stipules spinescent, 0.4–7 (–10) cm long, straight, slightly fusiform-inflated, fused basally and free for almost its entire length. Petiolar gland present. Leaf rhachis (0–) 1–5 (–9) cm long, a small gland usually at all or most of the pinna junctions. Pinnae 2–6 pairs, (1–) 1.5–4 (−7.2) cm long; leaflets 5–15 (–27) pairs per pinna, (2.8−) 3.5–8 (−12.5) × 1–2.5 (–5) mm. Inflorescence capitate, solitary or in fascicles; flowers bright yellow, fragrant; involucel basal. Pods brown, (4–) 5–10.5 (–21) × 0.5–0.7 (–1) cm, dehiscent, ± falcate, glabrous.

Native to Africa (Angola, Botswana, Lesotho, Mozambique, Namibia, South Africa, Swaziland, Zambia, Zimbabwe). It was introduced to Egypt in the 20th century as an ornamental tree (as recorded on herbarium sheets).

**Ecology and utilisation:** It is regarded as an environmental weed in Australia that can invade grasslands and rangelands and may exclude existing native vegetation in certain areas. In South Africa, this species is the most important woody invader of grazing lands and native grassland vegetation. Trees are important for bee farming as they indirectly result in the production of a pleasantly-flavoured honey [112,113]. In Egypt, the seeds were brought from the Australian Seed Center in 1999 as they have a high ability to fix nitrogen and thrive well in dry climates [114]. It is a fast-growing tree in the Northwest coast of Egypt and Sinai, also cultivated in some public and private gardens [45]. Additionally, the wood is used as fuel [38].

**Selected specimens:** Giza, Orman Garden, 12.071933, *s.coll. 1857* (CAIM); Private Garden, 14.11.2018, *R. Hassan 5036* (CAI); Mazhar Botanic Garden, 15.01.2019, *R. Hamdy 5037* (CAI).

**6. *Vachellia seyal*** var. *fistula* (Schweinf.) Kyal. and Boatwr., Bot. J. Linn. Soc. 172: 516 (2013).

Basionym: *Acacia fistula* Schweinf., Linnaea 35: 344 (1867-8); Delchev. Pl. exo. Egypte: 4 (1871); Delchev., Ap.Ge. végét. Exot. Égypte: 272 (1881); Delchev. Prom. et jard. Caire: 63 (1899). ≡ *Acacia seyal* var. *fistula* (Schweinf.) Oliv., Fl. Trop. Afr. 2: 351 (1871). Acacia flava f. fistula (Schweinf.) Roberty, Candollea 11: 145 (1948). *Acacia seyal* f. *fistula* (Schweinf.) Cufod., Bull. Jard. Bot. Etat Bruxelles 25, suppl.: 202 (1955).

Type: Sudan: Gedaref region, and Mountain Gule in the Sennar Province, Faschada, 24 Jan. 1869, *G. Schweinfurth 1084* (syntype: K-000244220!).

**Distinctive features:** Tree up to 17 m high. Bark glabrous, covered with greenish-yellow or orange-red powdery layer. Stipules spinescent, up to 8 cm long, straight, white, some pairs fused basally with bilobed whitish or greyish “ant-galls”. Petiolar gland present. Leaf rhachis 1–7.5 cm long, a small gland usually at the junction of the top 1 or 2 pinnae pairs. Pinnae (2–) 3–7 (–8) pairs, 1.5–4 cm long; leaflets (7–) 11–20 pairs per pinna, 3–8 (–10) × 1–1.5 (–3) mm. Inflorescence capitate, in fascicles; flowers bright yellow, fragrant; involucel basal. Pods brown, (5–) 7–22 × 0.5–0.9 cm, dehiscent, ± falcate, glabrous.

Native to Africa (Ethiopia, Malawi, Somalia, Sudan). Firstly-recorded in Egypt in the 19th century [43,115].

**Ecology and utilisation:** It is one of the most common trees in the savannah that occurs as a pure forest over quite large areas of the country. It grows in groups or patches of considerable size. The fistula is characteristic of the Nile region, tolerant to high pH, salts and periodic flooding. It is more tolerant to waterlogging than *A. seyal* var. *seyal*. The smoke produced by burning the wood acts as a fumigant against insects and lice. Chemicals in the bark kill the freshwater snails. Gum is eaten when fresh. The bark is extensively used as fodder during the dry season. The bark, leaves and gums are used for colds, diarrhoea, hemorrhage, headache and burns [116]. In Egypt, it had been cultivated in historical gardens as an ornamental tree in the past.

**Selected specimens:** Cairo, Rhoda Island, 26.11.1926, *G. Täckholm 5030* (CAI)- Giza, Zoological Garden, 5.06.1968, *M. El Mahdi 5031* (CAI).

**Figure 8:**
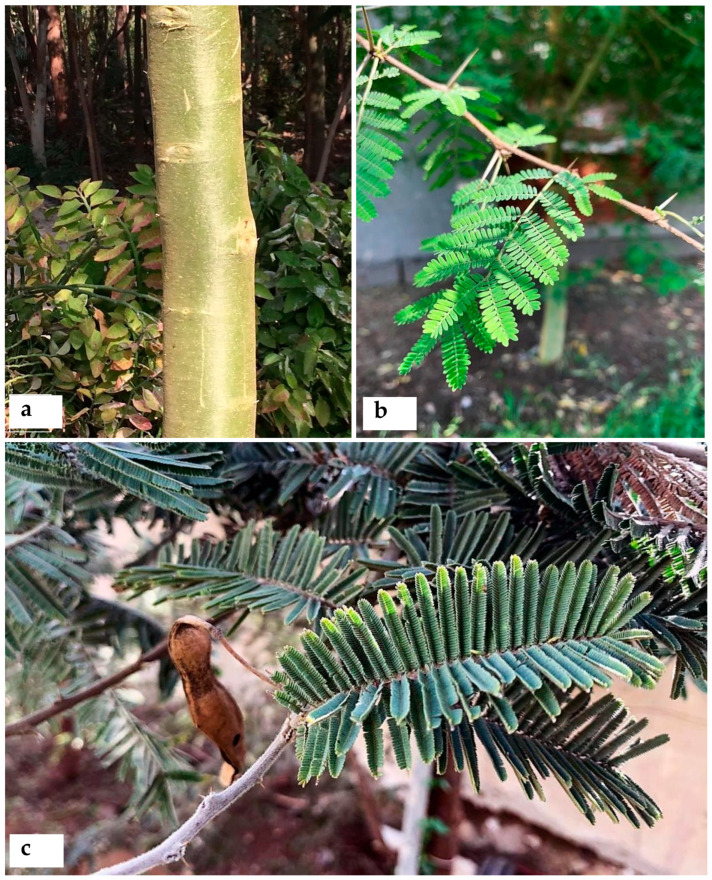
Examples of some studied *Vachellia* species. (**a**,**b**) *Vachellia xanthophloea,* (**a**) yellowish-green bark, (**b**) bipinnate leaves with spinescent straight stipules. (**c**) *Vachellia rehmanniana*, greyish-green, finely velvety pinnae, with mature brown dehiscent pod.

***7. Vachellia xanthophloea*** (Benth.) Banfi and Galasso, Atti Soc. Ital. Sci. Nat. Mus. Civico Storia Nat. Milano 149: 150 (2008, Jan.); P.J.H.Hurter, Mabberley’s plant book, ed. 3: 1021 (1 May, 2008), [Figure 8a,b]. Basionym: *Acacia xanthophloea* Benth., Trans. Linn. Soc. London 30 (3): 511 (1875); Bircher, Gard. Hesperides: 342 (1960).

**Type:** Southeast Tropical Africa: Malawi, Zomba [illegible], E end of Lake Shirwa, Oct. 1861, *Mello s.n.* (syntype: K-000244326!); Mozambique, Senna, 29 Dec. 1860, *J. Kirk s.n.* (syntype: K-000244328!); Mozambique, S. E. Chiloane, Aug. 1887, *L. Scott s.n.* (syntype: K-000244327!). = *Acacia songwensis* Harms, Bot. Jahrb. Syst. 30: 317 (1901).

**Distinctive features:** Tree up to 20 m high. Bark glabrous, powdery with a characteristic lime-green to greenish-yellow colour. Stipules spinescent, 1–8.5 cm long, straight. Petiolar gland present. Leaf rhachis 2.5–8 cm long, eglandular except at the junction of the top 1–2 pinnae pairs. Pinnae (1–) 3–6 (10) pairs, 0.3–3 cm long; leaflets 8–17 pairs per pinna, 2.5–6.5 × 0.5–1.75 mm. Inflorescence capitate, in fascicles; flowers bright yellow, fragrant; involucel in the middle. Pods yellowish-brown or brown, 3–14 × 0.7–1.4 cm, indehiscent, straight or slightly curved, irregularly constricted between seeds, glabrous.

Native to Africa (Kenya, Malawi, Mozambique, Somalia, South Africa, Swaziland, Tanzania, Zimbabwe). It was introduced to Egypt in the 20th century from South Africa, under the name “Fever tree” [38].

**Ecology and utilisation:** This tree is popular among birds for nest building as the thorns add extra protection against predators such as snakes. Young branches and leaves are eaten by elephants, while giraffes and monkeys eat the leaves and pods. Also, baboons eat the gum and green seeds. Medicinally the bark is used for treating fevers and eye complaints [117]. In Egypt, it is cultivated in gardens as an ornamental tree.

**Selected specimens:** Giza, Mazhar Botanic Garden, 15.01.2019, *R. Hamdy 5066* (CAI).

**Group B:** Leaves bipinnate; stipules spinescent, in pairs; flowers in round heads, yellowish-white, cream, white, rarely pale pink.
1A. Spines straight21B. Spines strongly hooked intermixed with straight or slightly recurved ones**9. *V. luederitzii* var. *luederitzii***2A. Leaves of mature shoots with 2–7 pairs of pinnae32B. Leaves of mature shoots with 8–44 pairs of pinnae43A. Leaflets width >1 mm; pods with conspicuous longitudinal veins**11. *V. robusta* subsp. *clavigera***3B. Leaflets width <1 mm; pods without longitudinal veins**8. *V. bellua***4A. Petiole and rachis densely hairy; pinnae closely arranged; involucel inconspicuous in the lower half of the peduncle**10. *V. rehmanniana***4B. Petiole and rachis moderately hairy; pinnae not closely arranged as above; involucel prominent in the upper half of the peduncle **12. *V. sieberiana* var. *woodii***

**8. *Vachellia bellula*** (Drake) Boatwr., Bot. J. Linn. Soc.179 (2):293 (2015). Basionym: *Acacia bellula* Drake, Bull. Mens. Soc. Linn. Paris 2: 1302–1303 (1897).

**Type:** Madagascar: Laolampia, *Grevé 62* (lectotype: P, designated by Du Puy and Villiers in Legum. Madagascar: 229, 2002).

**Distinctive features:** Shrub or small tree 2–11 m high. Bark fissured, silver-grey, with pendulous branches. Stipules spinescent, straight, persistent, 1–5 mm long. Rachis 2–3 mm long, with a minute gland between each pair of pinnae; pinnae 1–2 pairs, clustered at the nodes, 8–15 mm long; leaflets 4–10 pairs per pinna, minute, narrowly oblong, 1–3 (−4.5) mm long, fresh green. Inflorescence capitate, axillary, solitary, few-flowered, 12–13 mm in diameter; flowers creamy white. Pods pale grey, narrowly oblong, straight to slightly curved, 30–70 × (6–) 8–11 mm, flat, smooth, glabrous; margins straight to sinuous.

Native to Madagascar. It was introduced to Egypt in the 2000s [45].

**Utilisation:** In Egypt, it is cultivated as an ornamental tree in some gardens.

**Selected specimens:** Giza, Mazhar Botanic Garden, 15.01.2019, *R. Hamdy 5012* (CAI).

**9. *Vachellia luederitzii*** var. *luederitzii* (Engl.) Kyal. and Boatwr., Bot. J. Linn. Soc. 172 (4): 514 (2013). Basionym: *Acacia luederitzii* Engl., Bot. Jahrb. Syst. 10: 23, pl.3B (July 1888) pro parte.

**Type:** South West Africa: Namibia (Hereroland, Usakos), Otjimbingwe, alt. 1000 m. May 1886, *H. W. R. Marloth 1328* (lectotype: PRE-0423543-0!, designated by Ross and Brenan, 1967). = *Acacia goeringii* Schinz, Verh. Bot. Vereins Province Brandenburg 30: 239 (1888).

**Distinctive features:** Shrub or tree up to 15 m high. Bark rough, longitudinally fissured, grey- or reddish-brown to black. Stipules spinescent, short, strongly hooked, intermixed with long straight or slightly recurved spines. Petiolar glands absent. Leaf rhachis 0.7–4.8 cm long, eglandular or with small gland at the junction of the top 1–3 (–5) pinnae pair. Pinnae 3–9 (–13) pairs, 0.7–2.8 cm long; leaflets 10–26 pairs per pinna, 2–5 × 0.5–1.5 mm. Inflorescence capitate, solitary or in fascicles; flowers yellowish-white. Pods brown or reddish-brown to purplish, dehiscent, 3.2–13 × 1–2 cm, flat, straight, glabrous.

Native to Africa (Botswana, Namibia, South Africa, Zambia, Zimbabwe). It was introduced to Egypt in the 2000s [45].

**Ecology and utilisation:** Tree savannah, bush, scrub, thornveld, often associated with *A. erioloba* and often forming dense impenetrable thickets. A reddish-brown gum obtained from the stems is eaten. An infusion of the bark is used as an emetic to cleanse the body of various diseases. The brown wood is hard, heavy, tough and fire-resistant [118]. In Egypt, it is cultivated as an ornamental tree in some gardens.

**Selected specimens:** Giza, Mazhar Botanic Garden, 15.01.2019, *R. Hamdy 5039* (CAI).

**10. *Vachellia rehmanniana*** (Schinz) Kyal. and Boatwr., Bot. J. Linn. Soc.: 516 (2013), [Figure 8c].

Basionym: *Acacia rehmanniana* Schinz, Bull. Herb. Boiss. 6: 525 (1898).

**Type:** South Africa: Transvaal, Makapansberge-Streydpoort, *Rehmann 5517* (holotype: Z).

**Distinctive features:** Shrub or tree up to 12 m high. Bark rough, grey or reddish-brown to black. Stipules spinescent, 0.5–5.5 cm long, straight. Petiole densely hairy, petiolar gland present. Leaf rhachis 2.4–12 cm long, densely hairy, a small gland usually at the junction of the top 1–3 and bottom 1–6 pinnae pairs. Pinnae 15–44 pairs, closely arranged, 0.6–2.8 cm long; leaflets 24–48 pairs per pinna, 1–2.8 × 0.4–0.9 mm. Inflorescence capitate, solitary or in fascicles; flowers white or cream, fragrant; peduncle covered with dense hairs; involucel inconspicuous in the lower half. Pods grey- or reddish-brown to olive, (3–) 7–14 × 1–2.3 cm, dehiscent, straight, glabrous to pubescent.

Native to Africa (Botswana, South Africa, Zambia, Zimbabwe). It was introduced to Egypt in the 2000s [45].

**Utilisation:** In India, the wood is used to build temples and in ritual fires. The magical uses in South Africa are numerous. Burned wood stimulates psychic powers. It is both frost- and drought-resistant. An ideal plant for hedging/screening and attracting birds [119]. In Egypt, it is cultivated as an ornamental tree in some gardens.

**Selected specimens:** Giza, Private Garden, 14.11.2018, *R. Hassan 5055* (CAI); Mazhar Botanic Garden, 15.01.2019, *R. Hamdy 5056* (CAI).

**11. *Vachellia robusta*** subsp. *clavigera* (E. Mey.) Kyal. and Boatwr., Bot. J. Linn. Soc. 172: 516 (2013).

Basionym: *Acacia clavigera* E.Mey., Comm. Pl. Afr. Austr. 1: 168 (1836). ≡ *Acacia clavigera* subsp. *clavigera* Brenan, Kew Bull. 12: 367 (1958). *Acacia robusta* subsp. *clavigera* (E.Mey.) Brenan, Fl. Zambesiaca 3, 1: 104 (1970).

**Type:** South Africa, KwaZulu-Natal, near Durban, *J.F. Drège s.n.* (isotypes: K; MO-1889435!; P-00390990!, fragment).

**Distinctive features:** Tree up to 25 m high. Bark rough, fissured, grey to dark brown or blackish. Stipules spinescent, up to 1.2 cm long, straight. Petiolar gland prominent when present. Leaf rhachis 2–5 (−7.5) cm long, sparingly to densely pubescent, a small gland at the junction of the top 1–2 pinnae pairs. Pinnae 3–7 pairs, (1–) 2–5.5 (−7.5) cm long; leaflets (6–) 10–27 pairs per pinnae, (2.5−) 3.5–5.5 (–17) × 1.5–3.5 mm. Inflorescence capitate, usually fascicled; flowers white. Pods brown to dark reddish-brown, (2.5−) 6–15 (–22) × 1.2–1.7 cm, straight to falcate, glabrous with conspicuous longitudinal veins.

Native to Africa (Botswana, Malawi, Mozambique, Namibia, South Africa, Swaziland, Zambia, Zimbabwe). It was introduced to Egypt in the 2000s [45].

**Utilisation:** The wood is considered of rather poor quality but is sometimes used for making furniture, and shelving, although these uses are limited because of its strong tendency to warp. It is also used as a firewood. In traditional medicine, the powdered root is applied to swellings, and a decoction of the roots is used to treat dysmenorrhea [120]. In Egypt, it is cultivated as an ornamental tree in some gardens.

**Selected specimens:** Giza, Mazhar Botanic Garden, 15.01.2019, *R. Hamdy 5057* (CAI).

**12. *Vachellia sieberiana*** var. *woodii* (Burtt Davy) Kyal. and Boatwr., Bot. J. Linn. Soc. 172: 517 (2013), Figure 7c–e.

Basionym: *Acacia woodii* Burtt Davy, Bull. Misc. Inform. Kew 1922: 332 (1922).

**Type:** South Africa: Kwazulu-Natal, near Estcourt District, between Estcourt and Colenso, 1886, *J. Medley Wood 3528* (holotype: K; isotype: MEL-248971). *= lnga nefasia* Hochst. ex A.Rich., Tent. Fl. Abyss. 1: 237 (1847). *Acacia amboensis* Schinz, Mém. Herb. Boissier 1: 105 (1900). *Acacia monga* De Wild., Pl. Bequaert. 3: 62 (1925).

**Distinctive features:** Tree up to 25 m high. Bark coarse, yellowish-brown, peeling away in thin papery flakes. Stipules spinescent, 0.3–9 (−12.5) cm long, straight. Petiole moderately hairy, petiolar gland present. Leaf rhachis 2.5–13 cm long, moderately hairy, a small gland usually at the junction of the top 1–6 pinnae pairs. Pinnae (4–) 8–35 pairs; 0.8–4 (−6.5) cm long; leaflets 13–42 pairs per pinnae, 6–6.5 × 0.5–1.5 mm. Inflorescence capitate, solitary or in fascicles; flowers pale yellowish-white, involucel prominent in the upper half of the peduncle. Pods yellowish- or reddish-brown, 5–21 × 1.3–3.5 cm, dehiscent, straight or slightly curved, glabrous to densely pubescent.

Native to Africa (Angola, Botswana, Democratic Republic of Congo, Ethiopia, Namibia, Rwanda, South Africa, Sudan, Swaziland, Tanzania, Zimbabwe). It was introduced to Egypt in the 2000s and still growing in gardens [45].

**Ecology and utilisation:** It is found in woodland, wooded grassland and along riverbanks. It is a favourite nesting site for many birds. The pods have a musty scent and are eaten by cattle. In Central Africa, a bark/root decoction is used for inflammation of the urinary passages. Leaf, bark and resin are used as an astringent for colds/chest problems, diarrhoea, haemorrhage and eye inflammation. In Tanzania, bark is used to treat gonorrhea [121]. In Egypt, it is cultivated as an ornamental tree in some gardens.

**Selected specimens:** Giza, Mazhar Botanic Garden, 15.01.2019, *R. Hamdy 5064* (CAI).

**Group C:** Leaves bipinnate; stipules spinescent, swollen, straight to slightly reflexed near the apex, in pairs; flowers spicate.

**13. *Vachellia cornigera*** (L.) Seigler and Ebinger, Phytologia 87 (3): 153 (2005).

Basionym: *Mimosa cornigera* L., Sp. Pl. 1: 520 (1753). *≡ Acacia cornigera* (L.) Willd., Sp. Pl. ed. 4, 4 (2): 1080 (1806). *Tauroceras cornigerum* (L.) Britton and Rose, N. Amer. Fl. 23: 86 (1928).

**Type:** “Habitat in Mexico, Cuba.”, s.d., *G. Clifford s.n.* [from a cultivated plant grown in the garden of George Clifford III, Hartekamp Garden, between Haarlem and Leyden, Holland, collected by Linnaeus (No.4) and bearing his label ‘Mimosa cornigera’ presumably grown from Mexican seed, Herb. Cliff. 208.4] (lectotype: BM-000628753, designated by Rudd, 1964; isotype: US fragment and photo). = *Acacia spadicigera* Schltdl. and Cham., Linnaea 5 (4): 594–595 (1830); Walsingham, Hort. Rev. 56:67(1922). *Acacia campecheana* Schenck, Repert. Spec. Nov. Regni Veg. 12: 361 (1913). *Acacia cubensis* Schenck, Repert. Spec. Nov. Regni. Veg. 12: 360 (1913).

**Distinctive features:** Shrub or small tree up to 10 (15) m high. Bark shallowly furrowed, brown to grey. Stipules spinescent, light to dark reddish brown, 3–10 cm long, straight to slightly reflexed near the apex, stout and swollen near the base, glabrous to densely puberulent. Petiolar glands 1 (2). Pinnae 3–14 pairs per pinna, 3–7 cm long. Leaflets 15–40 pairs per pinna, oblong, 4–11 × 1.3–2.7 mm, obtuse mucronate at apex. Inflorescence spicate, axillary, solitary or in fascicles; spikes 20–35 × 8–11 mm; flowers sessile, pale yellow. Pods red to maroon, straight, oblong, 5–9 × 1.3–1.8 cm, coriaceous, indehiscent, glabrous to minutely puberulent.

Native to Central America (Belize, Costa Rica, El Salvador, Guatemala, Honduras, Mexico, Nicaragua). It was introduced to Egypt in the early 1920s from Jamaica [44].

**Ecology and utilisation:** Moist or dry thickets or thin forest, chiefly on the plains, forming dense thickets common in riparian and swamp habitats, mostly growing in fallow fields, pastures, roadsides and other disturbed sites. The pulp of the mature seedpod is commonly eaten. The bark contains tannins that treat diarrhoea and dysentery [122]. In Egypt, it was cultivated as an ornamental tree in some gardens in the past.

**Selected specimens:** Giza, Orman Garden, 8.07.1933, *F. Basta 0017036* (CAIM); Faculty of Agriculture, 21.04.1971, *M. Ezz El Din 5022* (CAI).

## 3. Materials and Methods

The scope of this study includes all species of the former broadly circumscribed genus *Acacia* that occur in Egypt.

The morphological investigation was based on the herbarium specimens kept in the following Egyptian herbaria: Cairo University (CAI), Agricultural Research Center (CAIM), Mazhar Botanical Garden (MAZHAR) and Orman Botanical gardens. Additionally, fresh specimens of cultivated *Acacia s.l.* species collected during many field trips conducted between 2018 and 2019 in most public Egyptian gardens and some private botanical gardens were examined. Reliable characters for species delimitation were observed (e.g., leaf structure, types of stipule, inflorescence, flower colour, pod characters). Species were identified with the help of digital photographs of the authentic specimens kept at virtual herbaria available on-line (WorldWideWattle database [4]; (K) Kew Royal Botanic Garden [123]; Tropicos [124]; the JSTOR Global Plants database [125]; (POWO) Plants of the World Online [126]). The identifications of Australian *Acacia s.s.* species were checked by consulting the free on-line WATTLE identification key [127], and the Flora of Australia Online (FOAO) treatments [128].

Identification keys for the studied genera, generic groups and species have been provided. The works of [13,26,30,129,130,131,132,133,134] are among the most useful contributions for our study.

Currently accepted name, type, accepted synonyms, distinctive features (based on our observations on the Egyptian plants and data collected from literature), origin, ecology (when available), utilisation and selected specimens were given for each studied taxon. Accepted names and synonyms were validated from the WorldWideWattle database [4]. Photographs of some living species found in this study were provided.

Abbreviations of taxon author and literature names follow those given in IPNI (International Plant Name Index) [135].

Type specimens were viewed as digital images obtained from JSTOR Global Plants database [125] or quoted from literature. Type terminology follows the International Code of Nomenclature [136]. Types that were seen by the authors are followed by (!).

The most commonly used synonyms in Egypt are included. The following symbols have been used for synonyms: = denotes a heterotypic synonym; ≡ denotes a homotypic synonym.

Selected voucher specimens from Egyptian herbaria were given with institution accession numbers when available. Acronyms of herbaria follow Index Herbariorum [137].

All *Acacia s.l.* names recorded in Egyptian taxonomic literature for indigenous and exotic species are included in Appendix A and Appendix B, respectively, with the currently accepted names. The exotic species are studied in some detail below. The species are arranged alphabetically under their respective group and numbered sequentially.

## Data Availability

The data presented in this study are available on request from the corresponding author.

## Appendix A

**Table A1:** Indigenous Species of *Acacia s.l.* Recorded in Egyptian Literature, with Currently Accepted Names.

Egyptian Literature	No. of Taxa	Name Used in Literature	Currently Accepted Name
Forsskål (1775)	1	*Mimosa nilotica* L.	*V. nilotica* (L.) P.J.H.Hurter & Mabb.
Delile (1813 & 1824)	5	*A. albida* Delile *A. gummifera* (Forssk.) Delile [Nom. nudum] *A. heterocarpa* Delile [unresolved] *A. nilotica* (L.) Willd. ex Delile *A. seyal* Delile	*Faidherbia albida* (Delile) A.Chev. *V. tortilis* subsp. *tortilis* (Forssk.) Galasso & Banfi *V. nilotica* subsp. *nilotica* (L.) P.J.H.Hurter & Mabb. *V. seyal* var. *seyal* (Delile) P.J.H.Hurter
Delchevalerie 1881	3	*A. albida* Delile *A. mellifera* Benth. *A. nilotica* Delile	*Faidherbia albida* (Delile) A.Chev. *S. mellifera* subsp. *mellifera* (Vahl) Seigler & Ebinger *V. nilotica* subsp. *nilotica* (L.) P.J.H.Hurter & Mabb.
Ascherson & Schweinfurth (1887)	6	*A. albida* Delile *A. arabica* var. *nilotica* (Forssk.) Asch. & Schweinf. *A. ehrenbergiana* Hayne *A. laeta* R.Br. ex Benth. *A. seyal* Delile *A. tortilis* (Forssk.) Hayne	*Faidherbia albida* (Delile) A.Chev. *V. nilotica* subsp. *nilotica* (L.) P.J.H.Hurter & Mabb. *V. flava* (Forssk.) Kyal. & Boatwr. *S. laeta* (R.Br. ex Benth.) Seigler & Ebinger *V. seyal* var. *seyal* (Delile) P.J.H.Hurter *V. tortilis* subsp. *tortilis* (Forssk.) Galasso & Banfi
Delchevalerie 1899	2	*A. arabica* var. *nilotica* Delile *A. mellifera* Hort.	*V. nilotica* subsp. *nilotica* (L.) P.J.H.Hurter & Mabb. *S. mellifera* subsp. *mellifera* (Vahl) Seigler & Ebinger **
Sickenberger (1901)	2	*A. ehrenbergiana* Hayne *A. tortilis* (Forssk.) Hayne	*V. flava* (Forssk.) Kyal. & Boatwr. *V. tortilis* subsp. *tortilis* (Forssk.) Galasso & Banfi
Muschler (1912)	6	*A. albida* Delile *A. ehrenbergiana* Hayne *A. laeta* R.Br. ex Benth. *A. arabica* var. *nilotica* (L.) Benth.^1^ *A. seyal* var. *seyal* Delile *A. tortilis* (Forssk.) Hayne	*Faidherbia albida* (Delile) A.Chev. *V. flava* (Forssk.) Kyal. & Boatwr. *S. laeta* (R.Br. ex Benth.) Seigler & Ebinger *V. nilotica* subsp. *nilotica* (L.) P.J.H.Hurter & Mabb. *V. seyal* var. *seyal* (Delile) P.J.H.Hurter *V. tortilis* subsp. *tortilis* (Forssk.) Galasso & Banfi
Walsingham (1922)	2	*A. laet*a R.Br. ex Benth. *A. seyal* Delile	*S. laeta* (R.Br. ex Benth.) Seigler & Ebinger *V. seyal* var. *seyal* (Delile) P.J.H.Hurter
Ramis (1929)	6	*A. albida* Delile *A. arabica* var. *nilotica* (Forssk.) Asch. & Schweinf. *A. ehrenbergian*a Hayne *A. laeta* R.Br. ex Benth. *A. seyal* var. *seyal* Delile *A. tortilis* subsp. *tortilis* (Forssk.) Hayne	*Faidherbia albida* (Delile) A.Chev. *V. nilotica* subsp. *nilotica* (L.) P.J.H.Hurter & Mabb. *V. flava* (Forssk.) Kyal. & Boatwr. *S. laeta* (R.Br. ex Benth.) Seigler & Ebinger *V. seyal* var. *seyal* (Delile) P.J.H.Hurter *V. tortilis* subsp. *tortilis* (Forssk.) Galasso & Banfi
Täckholm (1974)	15	*A. albida* Delile *A. asak* (Forssk.) Willd. *A. nilotica* subsp. *adansoniana* (Guill. et Perr.) Brenan *A. nilotica* subsp. *tomentosa* (Benth.) Brenan *A. ehrenbergiana* Hayne *A. etbaica* Schweinf. *A. gerrardii* subsp. *negevensis* Zohary *A. laet*a R.Br. ex Benth. *A. mellifera* (Vahl) Benth. *A. nilotica* (L.) Willd. ex Del. *A. nubica* Benth. *A. oerfota* (Forssk.) Schweinf. *A. raddiana* Savi *A. seyal* Delile *A. tortilis* (Forssk.) Hayne	*Faidherbia albida* (Delile) A.Chev. *S. asak* (Forssk.) Kyal. & Boatwr. *V. nilotica* subsp. *adstringens* (Schumach. & Thonn.) Kyal. & Boatwr. *V. nilotica* subsp. *tomentosa* (Benth.) Kyal. & Boatwr. *V. flava* (Forssk.) Kyal. & Boatwr. *V. etbaica* (Schweinf.) Kyal. & Boatwr. *V. gerrardii* subsp. *negevensis* (Zohary) Ragup., Seigler, Ebinger & Maslin *S. laeta* (R.Br. ex Benth.) Seigler & Ebinger *S. mellifera* subsp. *mellifera* (Vahl) Seigler & Ebinger *V. nilotica* subsp. *nilotica* (L.) P.J.H.Hurter & Mabb. *V. oerfota* var. *oerfota* (Forssk.) Kyal. & Boatwr. *V. oerfota* var. *oerfota* (Forssk.) Kyal. & Boatwr. *V. tortilis* subsp*. raddiana* (Forssk.) Galasso & Banfi (Savi) Kyal. & Boatwr. *V. seyal* var. *seyal* (Delile) P.J.H.Hurter *V. tortilis* subsp. *tortilis* (Forssk.) Galasso & Banfi
Boulos (2009)	12	*A. asak* (Forssk.) Willd. *A. ehrenbergiana* Hayne *A. etbaica* Schweinf. *A. laeta* R.Br. ex Benth. *A. mellifera* (Vahl) Benth. *A. nilotica* subsp. *nilotica* (L.) Delile *A. nilotic*a subsp. *tomentosa* Benth. *A. oerfota* var. *oerfota* (Forssk.) Schweinf. *A. pachyceras* O.Schwartz var. *najdensis* (Chaudhary) Boulos *A. seyal* Delile *A. tortilis* subsp. *tortilis* (Forssk.) Hayne *A. tortilis* subsp. *raddiana* (Savi) Brenan	*S. asak* (Forssk.) Kyal. & Boatwr. *V. flava* (Forssk.) Kyal. & Boatwr. *V. etbaica* (Schweinf.) Kyal. & Boatwr. *S. laeta* (R.Br. ex Benth.) Seigler & Ebinger *S. mellifera* subsp. *mellifera* (Vahl) Seigler & Ebinger *V. nilotica* subsp. *nilotica* (L.) P.J.H.Hurter & Mabb. *V. nilotica* subsp. *tomentosa* (Benth.) Kyal. & Boatwr. *V. oerfota* var. *oerfota* (Forssk.) Kyal. & Boatwr. *V. gerrardii* subsp. *negevensis* var. *najdensis* (Chaudhary) Ragup., Seigler, Ebinger & Maslin *V. seyal* var. *seyal* (Delile) P.J.H.Hurter *V. tortilis* subsp. *tortilis* (Forssk.) Galasso & Banfi *V. tortilis* subsp. *raddiana* (Savi) Kyal. & Boatwr.

Abbreviations: A. = Acacia, S. = Senegalia, V. = Vachellia; ^1^: nom. nudum = *Acacia arabica* Willd. var. *nilotica* (L.) Benth. Reference no.: Forsskål (1775) = [108]; Delile (1813) = [138]; Delile (1824) = [139]; Delchevalerie (1881) = [41]; Ascherson and Schweinfurth (1887) = [51]; Delchevalerie (1899) = [43]; Sickenberger (1901) = [115]; Muschler (1912) = [140]; Walsingham (1922) = [44]; Ramis (1929) = [141]; Täckholm (1974) = [36]; Boulos (2009) = [34].

## Appendix B

**Table A2:** Historical Data of All Exotic *Acacia s.l.* taxa Recorded in Egypt (Herbarium Specimens, Living Species and Species Just Recorded in Literature) with the Currently Accepted Names.

Name Used in Literature	Currently Accepted Name	CAI	CAIM	MAZHAR	Orman Herbarium	Private Botanical Gardens	Forsskål (1775)	Delile (1813 and 1824)	Delchevalerie (1871)	Delchevalerie (1881)	Ascherson and Schweinfurth (1887)	Delchevalerie (1899)	Sickenberger (1901)	Muschler (1912)	Walsingham (1922)	Ramis (1929)	Bircher (1960)
***A. abyssinica*** Hochst. ex Benth. [Basionym]	***V. abyssinica*** (Hochst. ex Benth.) Kyal. and Boatwr.										+						+
***A. acuminata*** Benth.	***A. acuminata*** Benth.																+
***A. adansonii*** Guill. and Perr. [Heterotypic synonym]	***V. nilotica*** subsp. ***adstringens*** (Schumach.) Kyal. and Boatwr.										+						+
***A. aestivalis*** E.Pritz	***A. aestivalis*** E.Pritz																+
***A. alata*** R.Br.	***A. alata*** R.Br.																+
***A. aneura*** F.Muell. ex Benth.	***A. aneura***^l^ F.Muell. ex Benth.	+		+	+										+		
***A. anisophylla*** S.Watson [Basionym]	***Senegalia × anisophylla*** (S.Watson) Britton and Rose																+
***A. armata*** R.Br. [Heterotypic synonym]	***A. paradoxa*** DC.				+				+	+		+			+		
***A. armata*** var. ***angustifolia*** Benth. [Heterotypic synonym]	***A. paradoxa*** DC.											+					
***A. armata*** var. ***microphylla*** Benth. [Heterotypic synonym]	***A. paradoxa*** DC.											+					
***A. ataxacantha*** DC. [Basionym]	***S. ataxacantha***^n,l^ (DC.) Kyal. and Boatwr.	+		+		+											
***A. auriculiformis*** A.Cunn. ex Benth.	***A. auriculiformis***^l^ A.Cunn. ex Benth.	+		+		+											+
***A. baileyana*** F.Muell.	***A. baileyana***^n,l^ F.Muell.	+		+	+	+											
***A. bellula*** Drake [Basionym]	***V. bellula***^n,l^ (Drake) Boatwr.	+		+		+											
***A. burkei*** Benth. [Basionym]	***S. burkei***^n,l^ (Benth.) Kyal. and Boatwr.	+		+		+											
***A. calamifolia*** Sweet ex Lindl.	***A. calamifolia***^l^ Sweet ex Lindl.	+		+		+						+			+		+
***A. campylacantha*** Hochst. ex A.Rich. [Basionym]	***S. polyacantha***^n,l^ subsp***. campylacantha*** (Hochst. ex A.Rich.) Kyal. and Boatwr.	+		+		+											
***A. catechu*** (L.f.) Willd. [Homotypic synonym]	***S. catechu***^l^ (L.f.) P.J.H.Hurter and Mabb.	+							+	+	+				+		
***A. cavenia*** (Molina) Hook. and Arn. [nom. illeg.]	***V. caven*** (Molina) Seigler and Ebinger	+							+	+	+						+
***A. concinna*** (Willd.) DC. [Homotypic synonym]	***S. rugata*** (Lam.) Britton and Rose														+		
***A. concurrens*** Pedley	***A. concurrens***^n,l^ Pedley	+		+		+											
***A. constricta*** var. ***paucispina*** Wooton and Standl. [Heterotypic synonym]	***V. constricta*** (Benth.) Seigler and Ebinger	+													+		
***A. cornigera*** (L.) Willd. [Homotypic synonym]	***V. cornigera*** (L.) Seigler and Ebinger	+	+		+												
***A. cultriformis*** A.Cunn. ex G.Don	***A. cultriformis***^l^ A.Cunn. ex G.Don			+	+	+			+	+		+					
***A. cuneate*** Benth. [Heterotypic synonym]	***A. truncata*** (Burm.f.) hort. ex Hoffmanns.											+					
***A. cyanophylla*** Lindl. [Heterotypic synonym]	***A. saligna*** (Labill.) H.L.Wendl.											+					+
***A. cyclops*** A.Cunn. ex G.Don	***A. cyclops*** A.Cunn. ex G.Don	+	+														+
***A. dealbata*** Link	***A. dealbata***^l^ Link			+	+	+			+		+	+			+		
***A. decipiens*** R.Br. [nom. illeg.]	***A. truncata*** (Burm.f.) hort. ex Hoffmanns.									+	+	+					
***A. decurrens*** Willd.	***A. decurrens*** Willd.									+	+				+		+
***A. decurrens*** var***. dealbata*** (Link) Muller [Orthographic variant]	***A. dealbata*** Link																+
***A. decurrens*** var. ***mollis sens.*** Benth. [Misapplied name]	***A. mearnsii*** De Wild.																+
***A. dentifera*** Benth.	***A. dentifera*** Benth.											+					
***A. discolor*** (Andrews) Willd. [Homotypic synonym]	***A. terminalis*** (Salisb.) J.F.Macrb.										+						
***A. dodoniafolia*** (Pers.) Willd.	***A. dodonaeifolia*** (Pers.) Balb.											+					
***A. drummondii*** Lindl. [Orthographic variant]	***A. drummondii*** Lindl.											+					
***A. eburnea*** (L.f.) Willd. [Homotypic synonym]	***V. eburnea*** (L.f.) P.J.H.Hurter and Mabb.											+			+		+
***A. elata*** A.Cunn. ex Benth.	***A. elata*** A.Cunn. ex Benth.																+
***A. elongata*** Sieber ex DC.	***A. elongata*** Sieber ex DC.									+					+		+
***A. erioloba*** E.Mey. [Basionym]	***V. erioloba***^n,l^ (E.Mey.) P.J.H.Hurter	+		+		+											
***A. excelsa*** Benth.	***A. excelsa*** Benth.											+			+		
***A. falcata*** Willd.	***A. falcata*** Willd.									+							
***A. farinosa*** Lindl.	***A. farinosa*** Lindl.								+			+					
***A. farnesiana*** (L.) Willd. [Homotypic synonym]	***V. farnesiana*** subsp. ***farnesiana*** ^l^ (L.)Wight and Arn.	+	+	+	+	+	+	+	+	+	+	+		+	+	+	+
***A. fimbriata*** A.Cunn.ex G.Don	***A. fimbriata***^n,l^ A.Cunn. ex G.Don	+		+	+	+											
***A. fistula*** Schweinf. [Basionym]	***V. seyal*** var. ***fistula*** ^l^ (Schweinf.) Kyal. and Boatwr.								+	+		+					
***A. galpinii*** Burtt Davy [Basionym]	***S. galpinii***^n,l^ (Burtt Davy) Seigler and Ebinger	+		+		+											
***A. giraffae sens. auct. mult.***	***V. erioloba*** (E.Mey.) P.J.H.Hurter																+
***A. glaucescens*** Willd. [nom. illeg. (superfluous)]	***A. binervia*** (J.C.Wendl.) J.F.Macbr.									+		+			+		
***A. glutinosa*** F.Muell. [Heterotypic synonym]	***A. verriculum*** R.S.Cowan and Maslin								+								
***A. goetzei*** Harms [Nom. cons.]	***S. goetzei*** subsp. ***goetzei*** ^n,l^ (Harms) Kyal. and Boatwr.			+		+											
***A. grandis*** Hort.	***A. pulchella*** var. ***glaberrima*** Meisn.											+					
***A. greggii*** A.Gray [Basionym]	***S. greggii*** (A.Gray) Britton and Rose	+	+														+
***A. harpophylla*** F.Muell. ex Benth.	***A. harpophylla*** F.Muell. ex Benth.	+			+												
***A. hastulata*** Sm.	***A. hastulata*** Sm.											+					
***A. homalophylla*** A.Cunn. ex Benth.	***A. homalophylla*** A.Cunn. ex Benth.														+		
***A. horrida*** (L.) Willd. [Homotypic synonym]	***V. horrida*** (L.) Kyal. and Boatwr.								+	+					+		
***A. inopinata*** Prain [Basionym]	***V. inopinata*** (Prain) Maslin, Seigler and Ebinger																+
***A. jennerae*** Maiden	***A. jennerae*** Maiden														+		
***A. juncifolia*** Benth.	***A. juncifolia*** Benth.														+		
***A. karroo*** Hayne [Basionym]	***V. karroo***^n,l^ (Hayne) Banfi and Galasso		+	+		+											
***A. koa*** A.Gray	***A. koa*** A.Gray														+		
***A. lahai*** Steud. and Hochst. ex Benth. [Basionym]	***V. lahai*** (Steud. and Hochst. ex Benth.) Kyal. and Boatwr.												+		+		
***A. lanigera*** A.Cunn.	***A. lanigera*** A.Cunn.														+		
***A. leptoclada*** A.Cunn. ex Benth.	***A. leptoclada*** A.Cunn. ex Benth.														+		
***A. leucophloea*** (Roxb.) Willd.	***V. leucophloea*** (Roxb.) Maslin, Seigler and Ebinger														+		
***A. leucophylla*** Hort.	***A. pendula*** A.Cunn. ex G.Don								+								
***A. linifolia*** (Vent.) Willd.	***A. linifolia*** (Vent.) Willd.									+		+			+		
***A. longifolia*** subsp. ***longifolia*** (Andrews) Willd.	***A. longifolia*** subsp. ***longifolia*** (Andrews) Willd.								+		+	+			+		+
***A. longissima*** H.L.Wendl.	***A. longissima*** H.L.Wendl.									+		+					
***A. luederitzii*** Engl. [Basionym]	***V. luederitzii*** var. ***luederitzii*** ^n,l^ (Engl.) Kyal. and Boatwr.	+		+		+											
***A. lunata*** Sieber ex DC. [Nom. illeg. (homonym)]	***A. lunata*** G.Lodd.									+							
***A. lutea*** (Mill.) Britton [Nom. rejec.]	***V. macracantha*** (Humb. and Bonpl. ex Willd.) Seigler and Ebinger																+
***A. macradenia*** Benth.	***A. macradenia*** Benth.																+
***A. mangium*** Willd.	***A. mangium***^n,l^ Willd.	+		+		+											
***A. marginata*** R.Br. [Heterotypic synonym]	***A. myrtifolia*** (Sm.) Willd.									+							
***A. mearnsii*** De Wild.	***A. mearnsii***^n,l^ De Wild.	+		+		+											
***A. melanoxylon*** R.Br.	***A. melanoxylon*** R.Br.	+			+				+	+	+	+					+
***A. modesta*** Wall. [Basionym]	***S. modesta***^l^ (Wall.) P.J.H.Hurter	+	+	+	+										+		
***A. moniliformis*** Griseb. [Heterotypic synonym]	***V. aroma*** (Gillies ex Hook. and Arn.) Seigler and Ebinger																+
***A. mollissima sens.*** Benth.	***A. mearnsii*** De Wild.																+
***A. mollissima*** Hort. ex Willd.	(Vent.) R.Br.								+		+						
***A. mollissima*** Hort. ex Willd. [ Heterotypic synonym]	***A. pubescens*** (Vent.) R.Br.								+	+	+						
***A. myrtifolia*** (Sm.) Willd.	***A. myrtifolia*** (Sm.) Willd.									+					+		
***A. nematophylla*** F.Muell. ex Benth.	***A. nematophylla*** F.Muell. ex Benth.											+					
***A. neriifolia*** A.Cunn. ex Benth.	***A. neriifolia***^l^ A.Cunn. ex Benth.	+	+	+											+		+
***A. nigrescens*** Oliv.[Basionym]	***S. nigrescens***^n,l^ (Oliv.) P.J.H.Hurter	+		+		+											
***A. obliqua*** A.Cunn. ex Benth. [Nom. illeg. (homonym)]	***A. acinacea*** Lindl.									+							
***A. oxycedrus*** Sieber ex DC.	***A. oxycedrus*** Sieber ex DC.											+					
***A. pendula*** A.Cunn. ex G.Don	***A. pendula*** A.Cunn. ex G.Don											+					
***A. pennata*** (L.) Willd. [Homotypic synonym]	***S. pennata*** subsp. ***insuavis*** ^n,l^ (Lace) Maslin	+		+		+											
***A. persiciflora*** Pax [Basionym]	***S. persiciflora***^n^ (Pax) Kyal. and Boatwr.	+															
***A. pervillei*** Benth.	***A. pervillei*** var. ***pervillei*** ^n,l^ Benth.			+		+											
***A. platyptera*** Lindl. [Basionym]	***A. alata*** var. ***platyptera*** (Lindl.) Meisn.											+					
***A. podalyriifolia*** A.Cunn. ex G.Don	***A. podalyriifolia*** A.Cunn. ex G.Don			+											+		+
***A. prominens*** A.Cunn. ex G.Don	***A. prominens*** A.Cunn. ex G.Don														+		
***A. pubescens*** (Vent.) R.Br.	***A. pubescens*** (Vent.) R.Br.	+			+					+							
***A. pycnantha*** Benth.	***A. pycnantha*** Benth.	+													+		
***A. rehmanniana*** Schinz [Basionym]	***V. rehmanniana***^n,l^(Schinz) Kyal. and Boatwr.	+		+		+											
***A. retinodes*** Schltdl.	***A. retinodes***^l^ Schltdl.	+	+	+	+	+			+			+					
***A. riceana*** Hensl.	***A. riceana*** Hensl.											+					
***A. richii*** A.Gray	***A. richii*** A.Gray																+
***A. robusta*** Burch. [Basionym]	***V. robusta*** subsp. ***clavigera*** ^n,l^ (E.Mey.) Kyal. and Boatwr.	+		+		+											
***A. rubida*** A.Cunn.	***A. rubida*** A.Cunn.																+
***A. salicina*** Lindl.	***A. salicina*** Lindl.		+												+		
***A. saligna*** (Labill.) H.L.Wendl.	***A. saligna***^l^ (Labill.) H.L.Wendl.	+	+	+	+				+						+		+
***A. scleroxylon*** Tussac. [Orthographic variant]	***Parasenegalia skleroxyla*** (Tussac) Seigler and Ebinger																+
***A. senegal*** var. ***rostrata*** Brenan [Basionym]	***S. senegal*** subsp***. rostrata*** ^n,l^ (Brenan) Kyal. and Boatwr.	+				+											
***A. senegal*** (L.) Willd. [Homotypic synonym ]	***S. senegal*** subsp. ***senegal*** (L.) Britton	+	+								+				+		+
***A. sentis*** F.Muell. ex Benth. [nom. illeg. (superfluous)]	***A. victoriae*** Benth.			+													+
***A. sieberiana*** DC.	***V. sieberiana*** var. ***woodii*** ^n,l^ (Burtt Davy) Kyal. and Boatwr.	+		+		+											
***A. sophorae*** (Labill.) R.Br. [Homotypic synonym]	***A. longifolia*** subsp. ***sophorae*** ^l^ (Labill.) Court			+					+	+							
***A. spadicigera*** Schltdl. and Cham. [Heterotypic synonym]	***V. cornigera*** (L.) Seigler and Ebinger														+		
***A. spectabilis*** A.Cunn. ex Benth.	***A. spectabilis*** A.Cunn. ex Benth.									+					+		
***A. sphaerocephala*** Schltdl. and Cham. [Basionym]	***V. sphaerocephala*** (Schltdl. and Cham.) Seigler and Ebinger																+
***A. spirocarpa*** var. ***major*** Schweinf. [Heterotypic synonym]	***V. tortilis*** subsp. ***spirocarpa*** (Hochst. ex. A.Rich.) Kyal. and Boatwr.											+					
***A. spirocarpa*** var. ***minor*** Schweinf. [Heterotypic synonym]	***V. tortilis*** (Forssk.) Galasso and Banfi											+					
***A. stricta*** (Andrews) Willd.	***A. stricta*** (Andrews) Willd.									+							
***A. suaveolens*** (Sm.) Willd.	***A. suaveolens*** (Sm.) Willd.											+					+
***A. suma*** (Roxb.) Kurz [Isonym]	***S. polyacantha*** subsp. ***polyacantha*** (Willd.) Seigler and Ebinger														+		+
***A. sundra*** (Roxb.) DC. [spelling variant]	***A. chundra*** (Rottler) Willd.														+		
***A. trinervata*** Sieber ex DC.	***A. trinervata*** Sieber ex DC.											+					
***A. tucumanensis*** Griseb. [Basionym]	***S. tucumanensis*** (Griseb.) Seigler and Ebinger																+
***A. verugera*** Schweinf. [Heterotypic synonym]	***V. sieberiana*** (DC.) Kyal. and Boatwr.										+						
***A. verticillata*** (L’Hér.) Willd.	***A. verticillata*** (L’Hér.) Willd.								+	+		+			+		
***A. vestita*** Ker Gawl.	***A. vestita*** Ker Gawl.				+					+		+					
***A. victoriae*** Benth.	***A. victoriae***^n,l^ Benth.	+		+		+											
***A. villosa*** (Sw.) Willd. [Homotypic synonym]	***Acaciella villosa*** (Sw.) Britton and Rose																+
***A. visco*** Lorentz ex Griseb. [Basionym]	***Parasenegalia visco*** (Lorentz ex Griseb.) Seigler and Ebinger														+		
***A. xanthophloea*** Benth. [Basionym]	***V. xanthophloea***^l^ (Benth.) Banfi and Galasso			+		+											+

Abbreviations: CAI = Cairo University Herbarium, CAIM = Agricultural Museum Herbarium, A. = Acacia, S. = Senegalia, V. = Vachellia, ^n^: newly recorded, ^l^: living.

**Excluded Species**

***A. fruticosa*** Mart., Flora 20(2): 107 (1837); Bircher, Gard. Hesperides 339 (1960). [Heterotypic synonym] = *Piptadenia adiantoides* (Spreng.) J.F.Macbr., Contr. Gray Herb. 59: 17 (1919).

***A. glauca*** Willd., Sp. Pl., ed. 4 [Willdenow] 4(2): 1075 (1806); Delchev. Pl. Exo. Egypte: 4, no.10 (1871) [nom. illeg. (homonym)] = *Leucaena leucocephala* (Lam.) de Wit, Taxon 10: 53 (1961).

***A. houstonii*** (L’Hér.) Willd., Sp. Pl., ed. 4 [Willdenow] 4: 1062 (1806); Delchev. Prom. et jard. Caire: 63 (1899) [Homotypic synonym] ≡ *Calliandra houstoniana* (Mill.) Standl. var. *houstoniana*, Contr. U.S. Natl. Herb. 23: 386 (1922).

***A. inermis*** Hort. ex Steud., Nom. Bot. 688 (1840); Delchev. Pl. Exo. Egypte: 4, no.13 (1871) [Heterotypic synonym] = *Gleditsia triacanthos* L., Sp. Pl. 2: 1056 (1753).

***A. julibrissin*** (Durazz.) Willd., Sp. Pl., ed. 4 [Willdenow] 4(2): 1065 (1806); Delchev. Prom. et jard. Caire: 63 (1899) [Homotypic synonym] ≡ *Albizia julibrissin* Durazz Mag. Tosc. 3(4): 11 (1772).

***A. lebbek*** (L.) Willd., in J.M. Lock and J. Heald, Legumes Indo-China 37 (1994); Forssk., Fl. Aegypt-Arab.: [LXXVII no.552] (1775); Delile, Descr. Égypte, Hist. Nat. 2(1): 79 (1813); Delchev.Pl. Exo. Egypte: 4, no.14 (1871); Delchev. Prom. et jard. Caire: 63 (1899). [Orthographic variant] =*Albizia lebbeck* (L.) Benth., London J. Bot. 3: 87 (1844).

***A. leucocephala*** (Lam.) Link, Enum. Hort. Berol. Alt 2: 444–445 (1822); Delchev. Prom. et jard. Caire: 63 (1899); [Homotypic synonym] ≡ *Leucaena leucocephala* (Lam.) de Wit, Taxon 10: 53 (1961).

***A. lophantha*** Willd., Sp. Pl., ed. 4 [Willdenow] 4(2): 1070 (1806); Delchev., Pl. Exo. Egypte: 4, no.17 (1871); Delchev. Prom. et jard. Caire: 63 (1899) [Basionym] ≡ *Paraserianthes lophantha* (Willd.) I.C. Nielsen, Bull. Mus. Natl. Hist. Nat., B, Adansonia 5(3): 326 (1983).

Unresolved names

***A. decurrens*** (Vent.) Willd. var. ***mollissima*** (Willd.) Asch. and Schweinf., Asch. and Schweinf., Ill. Fl. Égypte, Mém. Inst. Égypt. 2: 72 (1887).

***A. lophantha*** var. ***neumannii*** Hort., Delchev. Prom. et jard. Caire: 63 (1899).

*A. verticillata* var. robusta hort. ex L. Neumann, Delchev. Prom. et jard. Caire: 64 (1899).

***A. verek*** Guill. and Perr., in A. Guillemin et al., Fl. Seneg. Tent. 1: 245 (1832); Asch. and Schweinf., Ill. Fl. Égypte, Mém. Inst. Égypt. 2: 72 (1887); Walsingham, Hort.Rev.56:67(1922). [nom. illeg. (superfluous), mentioned by Ross (1979)].

**Unvalidated names**

***A. acanthocarpa***(no author given) [Mexico], Walsingham, H.R.: 106 (1922), it could be *A. acanthocarpa* Willd. as recorded in the list of seeds available for exchange (Anonymous, 1922), Enum. Pl. 1057 (1809), heterotypic synonym = *Mimosa aculeaticarpa* Ortega, Nov. Pl. Descr. Dec. 10: 134 (1800).

***A. adinocephala*** (no author given) = *Pithecolobium adinocephalum* [Costa Rica, and Panama to British Honduras], Bircher, Gard. Hesperides: 336 (1960). This is possibly in error for *Albizia adinocephala* (Donn. Sm.) Britton and Rose ex Record. Trop. Woods 10: 22 (1927).

***A. albicans*** Hort (no author given) [New Holland], Delchev. Pl. Exo. Egypte: 3, no. 1(1871); Delchev. Prom. et jard. Caire: 63 (1889). It could be *A. albicans* Kunth, Mimoses 87, pl. 27 (1821), basionym = *Havardia albicans* (Kunth) Britton and Rose, N. Amer. Fl. 23: 41 (1928).

***A. buxifolia*** (no author given) [Australia], Waslingham, H.R.: 106 (1922).

***A. coccinea*** Hort (no author given) [New Holland], Delchev. Prom. et jard. Caire: 63 (1889), Could be *A. buxifolia* subsp. *buxifolia* A. Cunn.

***A. dichocarpa*** Hort (no author given) [New Holland], Delchev. Pl. Exo. Egypte: 4, no.6 (1871).

***A. drummundi*** Benth. [New Holland], Delchev. Prom. et jard. Caire: 63 (1889), Could be *A. drummondii* Lindl.

***A. juniperina*** DC. [New Holland], Delchev. Prom. et jard. Caire: 63 (1889), could be *A. ulicifolia* (Salisb.) Court.

***A. latifolia*** Willd., Delchev. Prom. et jard. Caire: 63 (1889).

***A. leiophylla*** Hort (no author given) [New Holland], Delchev. Prom. et jard. Caire: 63 (1889).

***A. namsi*** Willd. [China], Delchev. Prom. et jard. Caire: 63 (1889).

***A. oleifolia***, Hort (no author given) [New Holland], Delchev. Pl. Exo. Egypte: 4, no.21 (1871). It could be *A. oleifolia* A. Cunn. ex Loudon (1830), nom. nudum = *A. uncinata* Lindl. (Australia).

***A***. ***rosea*** Hort (no author given) [Virginia], Delchev. Pl. Exo. Egypte: 4, no.24 (1871).

***A. sp***.? du Sennar [Africa: Sennar], Delchev. Pl. Exo. Egypte: 4, no. 26 (1871).

***A. ulicina*** Labill. [Van Diemen], Delchev. Pl. Exo. Egypte: 4, no. 28 (1871).

***A. xylophylloides*** Hort [New Holland], Delchev. Prom. et jard. Caire: 64 (1889).
